# Mucoromycotina Fungi Possess the Ability to Utilize Plant Sucrose as a Carbon Source: Evidence From *Gongronella* sp. w5

**DOI:** 10.3389/fmicb.2020.591697

**Published:** 2021-01-13

**Authors:** Xiaojie Wang, Junnan Fang, Pu Liu, Juanjuan Liu, Wei Fang, Zemin Fang, Yazhong Xiao

**Affiliations:** ^1^School of Life Sciences, Anhui University, Hefei, China; ^2^Anhui Key Laboratory of Modern Biomanufacturing, Hefei, China; ^3^Anhui Provincial Engineering Technology Research Center of Microorganisms and Biocatalysis, Hefei, China; ^4^College of Horticulture, Anhui Agricultural University, Hefei, China

**Keywords:** plant–fungi interaction, invertase, sucrose transporter, Mucoromycotina, *Gongronella* sp. w5

## Abstract

Mucoromycotina is one of the earliest fungi to establish a mutualistic relationship with plants in the ancient land. However, the detailed information on their carbon supply from the host plants is largely unknown. In this research, a free-living Mucoromycotina called *Gongronella* sp. w5 (w5) was employed to explore its effect on *Medicago truncatula* growth and carbon source utilization from its host plant during the interaction process. W5 promoted *M. truncatula* growth and caused the sucrose accumulation in *M. truncatula* root tissue at 16 days post-inoculation (dpi). The transportation of photosynthetic product sucrose to the rhizosphere by *M. truncatula* root cells seemed accelerated by upregulating the SWEET gene. A predicted cytoplasmic invertase (*GspInv*) gene and a sucrose transporter (*GspSUT1*) homology gene in the w5 genome upregulated significantly at the transcriptional level during w5–*M. truncatula* interaction at 16 dpi, indicating the possibility of utilizing plant sucrose directly by w5 as the carbon source. Further investigation showed that the purified GspInv displayed an optimal pH of 5.0 and a specific activity of 3380 ± 26 U/mg toward sucrose. The heterologous expression of *GspInv* and *GspSUT1* in *Saccharomyces cerevisiae* confirmed the function of GspInv as invertase and GspSUT1 as sugar transporter with high affinity to sucrose *in vivo*. Phylogenetic tree analysis showed that the ability of Mucoromycotina to utilize sucrose from its host plant underwent a process of “loss and gain.” These results demonstrated the capacity of Mucoromycotina to interact with extant land higher plants and may employ a novel strategy of directly up-taking and assimilating sucrose from the host plant during the interaction.

## Introduction

Plants co-exist with fungi to adapt to terrestrial environments starting from the dawn of life on land (Berbee et al., 2017; Martin et al., 2017). Glomeromycotina was previously considered the earliest fungi (dating back to ca. 460 million years ago, Ma) to form symbiotic relationships with plants based on their genomic data and characteristic arbuscule-like structures in fossil plants (Parniske, 2008; Desiro et al., 2015). However, this paradigm was challenged by new lines of evidence from molecular, cytological, functional, and paleontological studies (Bidartondo et al., 2011; Field et al., 2015). Some fungi from Mucoromycotina have been evaluated as originating contemporaneously with Glomeromycotina (James et al., 2006; Spatafora et al., 2016; Bonfante and Venice, 2020). Strullu-Derrien et al. (2014) recently proved the existence of Mucoromycotina in the fossils of early vascular plants from a 407 (Ma) Rhynie Chert, placing Mucoromycotina as potentially key players in the initial colonization of the Earth’s landmasses.

Compared with Glomeromycotina, which is a strict obligate biotroph with organic carbon coming entirely from living plants (Field et al., 2019), some fungi from Mucoromycotina could be aseptically cultured and may be facultative saprotrophs that could obtain nutrients independently from soil organic matter (Lindahl and Tunlid, 2015). The ability of Mucoromycotina to keep a symbiotic relationship with plants has likely undergone several “losses and gains” through land plant evolution (Hoysted et al., 2018; Rimington et al., 2019) and are likely limited to non-vascular plants and early terrestrial vascular plants (Rimington et al., 2015; Field et al., 2019). Limited knowledge was gained on the molecular basis for the functioning of Mucoromycotina fungal partnerships in non-vascular and vascular plants. Investigations on the symbiotic relationship between Mucoromycotina and higher plants are also in the primary stage (Field et al., 2015, 2019; Hoysted et al., 2018). Only few ferns (such as *Ophioglossum* and *Osmunda*) and Lycopodiaceae (such as *Lycopodiella inundata*) have been experimentally confirmed to exchange substances with Mucoromycotina fungi, such as *Endogone* and *Sphaerocreas* (Field et al., 2012; Desiro et al., 2017; Hoysted et al., 2018). Whether Mucoromycotina could form a symbiotic relationship with extant land higher plants needs further investigation.

Fatty acids have been recently confirmed to be transported from the host to the arbuscular mycorrhizal fungi (AM fungi) during fungus–plant symbiosis (Jiang et al., 2017; Luginbuehl et al., 2017). Meanwhile, the plant host also provides sugars, predominantly sucrose, and its hydrolysates, as nutrients to support the growth of fungal cells. In some cases, plants could allocate up to 30% of their photosynthate carbon sucrose to mycorrhizal fungi (Doidy et al., 2012; Behie and Bidochka, 2014; Ruan, 2014). Plant sucrose transporters (SUTs), monosaccharide transporters (MSTs), cell-wall invertases (CWInv), and SWEET (Sugars Will Eventually be Exported Transporter) proteins are involved in the utilization of plant sugars by the beneficial symbiotic fungus inhabited in the rhizosphere (An et al., 2019; Wipf et al., 2019). For example, the AM fungi could not uptake sucrose directly from host plants. They receive glucose hydrolyzed from sucrose by plant CWInvs through plant SWEETs or MSTs (Ruan, 2014; Roth and Paszkowski, 2017; Wipf et al., 2019). For example, An et al. (2019) found that leguminous model plant *Medicago truncatula* SWEET transporter MtSWEET1b (Medtr3g089125) transports glucose across the peri-arbuscular membrane to maintain arbuscules for a healthy mutually beneficial symbiosis. SUTs mainly play roles in carbon partitioning rather than direct sucrose supply to the fungus in mycorrhizal roots (Gaude et al., 2012; Wipf et al., 2019). However, some beneficial endophytic fungi, such as *Trichoderma virens*, could directly utilize plant sucrose by special Suc/H^+^ co-transporter (TvSUT)-like plant SUT proteins and invertases (Blee and Anderson, 2002; Vargas et al., 2009). Advances in genome sequencing technologies resulted in discovering that the overall access of fungi to plant sucrose appears to be determined by the Glycoside Hydrolase 32 (GH32) family gene in the fungal genome during fungus–plant interaction (Parrent et al., 2009). The number of GH32 in a species is related to its ecological strategy (Parrent et al., 2009). For example, plant pathogens such as *Fusarium oxysporum*, *Fusarium verticillioides*, and *Nectria haematococca* typically show GH32 family expansions, and their enzymes play critical roles in pathogen nutrition (Parrent et al., 2009). Within plant beneficial fungi, the so-far characterized AM fungi lacked GH32 invertases (Wipf et al., 2019). Genomic analysis of several ectomycorrhizal (ECM) fungi, such as *Laccaria bicolor*, also showed that mycorrhizal fungi lack genes encoding invertases, suggesting their dependence on the glucose released by its green host (Martin et al., 2008). However, what kind of carbon source Mucoromycotina fungi obtain from their host plant and how they get their hosts’ carbon source remain unclear.

*Gongronella* species are non-pathogenic, soil-borne free-living Mucoromycotina fungi (Hoffmann et al., 2013). They are ubiquitous and prolific within many ecosystems (Rodrigues et al., 2014; Li et al., 2016; You et al., 2017; Cruz-Lachica et al., 2018). In Waipara (1997) reported that *Gongronella* sp. was one of the important root colonizing fungi of pasture roots. *Gongronella* sp. w5 (w5) is a fungus sampled from the topsoil of Mount Shushan in Hefei, Anhui Province, China (Wei et al., 2010). This strain promotes improvement in the growth of several plants, such as *Actinidia chinensis* (Dong et al., 2018). Moreover, w5 prefers sucrose to glucose and fructose as the carbon source (Hu et al., 2019), leading to the hypothesis that w5 may take sucrose from its host plant. In the present study, *M. truncatula* root was inoculated with w5 because the former is a model plant of legumes that originated from a common ancestor 60 Ma (Young et al., 2011) and could form symbiotic relationships with various AM fungi. The results showed that w5 inoculation caused sucrose accumulation in the *M. truncatula* root tissues. W5 promoted *M. truncatula* growth and may directly utilize the sucrose of the host plant using cytoplasm invertase GspInv and SUT GspSUT1 during its interaction with *M. truncatula*. The findings provide new insight into the interaction between Mucoromycotina and host plants in the rhizosphere.

## Materials and Methods

### Strains, Vectors, and Reagents

*Gongronella* sp. w5 (China Center for Type Culture Collection No. AF2012004) was maintained on potato dextrose agar slants at 4°C (Pan et al., 2014). *Saccharomyces cerevisiae* SEY2102 was purchased from Beijing Beina Science and Technology Co., Ltd. (Beijing China). The expression vector pDR195 was purchased from Hunan Fenghui Biotechnology Co., Ltd. (Hunan, China). *S. cerevisiae* EBY.VW4000 and plasmid pPMA1 were presented by Dr. Zhiyong Pan of Huazhong Agricultural University. All other chemicals and reagents were of analytical grade unless otherwise indicated.

### Cultivation of Fungal Strain and Plant Bioassays

**FIGURE 1:**
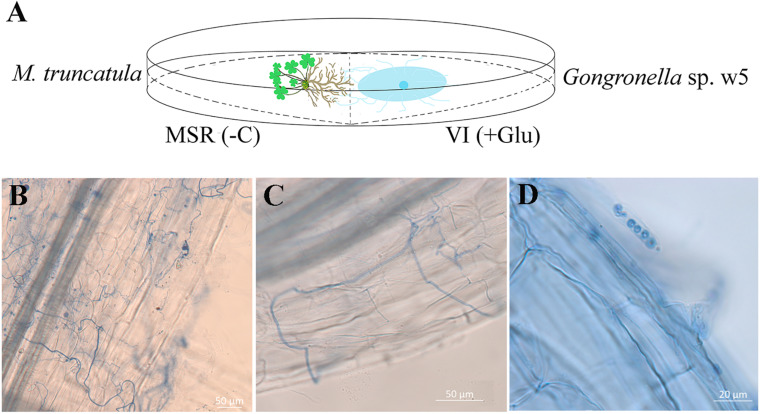
**(A)** Schematic representation of the medium and light microscope of w5 grows with the *M. truncatula* root. MSR(–C), MSR medium without carbon source. VI(+Glu), VI medium with glucose as carbon source. **(B,C)** w5 hypha around *M. truncatula* roots. **(D)** w5 spores in the hair of *M. truncatula* roots.

The agar plates used to evaluate the effect of w5 inoculation on *M. truncatula* A17 growth were prepared as follows: 10 ml of VI medium (with glucose as the carbon source) (Pan et al., 2014) was poured into a Petri dish to make an agar slant that occupies half the space of a plate (90 mm in diameter). After its solidification, the other 10 ml of the Modified Strullu–Romand (MSR) medium (without glucose) (Voets et al., 2008) was poured into the other half-space of the plate (Figure 1A). The *M. truncatula* seeds were surface-disinfected via immersion into 30% sodium hypochlorite solution for 15 min, rinsed three times with sterile water, and then planted in the MSR medium in Petri dishes (three seeds per plate). W5 mycelial plug (5 mm in diameter) was inoculated on the VI medium (Figure 1A). W5 grown alone on VI medium and *M. truncatula* grown alone on MSR were used as the controls. They were incubated in an illumination incubator (16/8 h light/dark photoperiod, 25°C). The w5 mycelia closely contacted with plant roots were withdrawn independently at 6 and 16 days post-inoculation (dpi) for quantitative reverse transcription PCR (qRT-PCR) analysis. The plant root samples contacted with w5 mycelia were also withdrawn at 16 dpi and stored at −80°C for metabolomic, transcriptomic, and qRT-PCR analysis. The chlorophyll concentration of plants after w5 inoculation was assayed as a soil and plant analyzer development (SPAD) value using SPAD-502 (Minolta, Japan) (Liu et al., 2010). The physical parameters of the leaf and root parts of *M. truncatula* after 16 days of cultivation were measured using the image analysis WinRHIZO system (Wang and Zhang, 2009). The samples were dried to a constant weight at 80°C. Observation of w5 on *M. truncatula* roots refers to Phillips and Hayman (1970). The infection frequency (F%) of w5 in the root system was counted and calculated as F% (= the number of segments infected by w5/the number of segments × 100%).

### Metabolomics and Quantification of Sugars in *M. truncatula* Root Tissue

The *M. truncatula* root tissue (500 mg) was ground using liquid nitrogen. The metabolites in the root were then extracted with 400 μl of extraction solution (methanol:acetonitrile:water = 4:4:2, v/v) for 60 min at −20°C. After centrifugation at 14,000 × *g* for 20 min, the compounds in the supernatant were withdrawn by drying in a vacuum, re-dissolved in 100 μl acetonitrile solution (acetonitrile:water = 1:1, v/v), and used for mass spectrometry analysis. In brief, the compounds were analyzed using Agilent 1290 Infinity UHPLC equipped with ACQUITY UPLC BEH Amide chromatographic column (Waters, 1.7 μm, 2.1 mm × 100 mm) at 25°C. The mobile phase consisted of 25 mM ammonium acetate and 25 mM acetic acid in water and 100% acetonitrile. The gradient of the latter increased linearly from 0 to 95% (v/v) at 1 min, to 65% at 14 min, and to 40% at 16 min, and then kept at 40% until 18 min; it increased to 95% at 18.1 min and kept at 95% until 23 min, with a flow rate of 0.3 ml/min (Plumb et al., 2005).

The compounds separated in columns were identified using a Triple TOF 6600 mass spectrometer (AB SCIEX). The electrospray ionization (ESI) source conditions are listed as follows: ion source gas1 was 60, ion source gas2 was 60, and curtain gas was 30. The source temperature was 600°C. The IonSpray Voltage Floating field was ±5500 V in positive and negative ionization modes. The TOF-MS scan m/z range and accumulation time were 60–1000 Da and 0.20 s/spectrum. The product ion scan m/z range was 25–1000 Da. The product ion scan accumulation time was 0.05 s/spectrum. The second mass spectrometry was conducted through information-dependent acquisition (IDA) based on high sensitivity mode, with declustering potential and collision energy set as ±60 V (negative ionization mode) and 35 ± 15 eV, respectively. The IDA settings are as follows: the isotopes within 4 Da were excluded, and the number of candidate ions for monitoring per cycle was six (Ivanisevic et al., 2013; Benton et al., 2015).

The metabolic information was mapped to the Kyoto Encyclopedia of Genes and Genomes (KEGG)^1^ to identify enriched KEGG pathways. Enrichment analysis was performed on OmicsBean^2^ to explore the effects of differentially accumulated metabolites and identify the internal relationships between them. Only pathways with *P* values < 0.05 were considered to have a significant enrichment. All experiments were designed with five replicates.

Sucrose, fructose, and glucose concentrations in roots were quantified using high-performance liquid chromatography (HPLC) with evaporative light scattering detection (ELSD). The analysis was carried out on a TSKgel Amide-80 column (4.6 mm × 25 cm, 5 μm) with isocratic elution of acetonitrile:water (75:25, v/v). The drift tube temperature of the ELSD system was set to 80°C, and the nitrogen flow rate was 1.6 L/min.

### Transcriptome Analysis

Three biological replicates of *M. truncatula* root with or without w5 inoculation were used for the transcriptome analysis. The total RNA of the root samples was extracted using the TransZol Plant RNA Kit (TransGen, Beijing, China). Paired-end cDNA sequencing libraries were carried out using Illumina TruSeq Stranded Total RNA Library Preparation Kit following the manufacturer’s protocol. Libraries were sequenced on the Illumina HiSeq 2000 platform (Shanghai Personal Biotechnology Co., Ltd.) (Wang et al., 2018). Gene assembly was referenced to the *M. truncatula* genome database^3^ (Krishnakumar et al., 2015). The assembled genes were annotated by BLAST searches against the NCBINr^4^, SwissProt^5^, COG^6^, and KEGG databases and MapMan binning (Thimm et al., 2004). Gene Ontology (GO) assignment was conducted to classify the function of *M. truncatula* transcripts. Principal component analysis (PCA) was analyzed on R tool 2.12.0 (Stacklies et al., 2007).

### qRT-PCR Analysis

W5 mycelia cultured in liquid VI medium, with 2% sucrose as the sole carbon source, were harvested after 24 h of cultivation and used for qRT-PCR analysis to determine the transcription of target genes involved in sucrose metabolism. Total RNA was extracted using the RNAprep Pure Plant Kit (Tiangen, Beijing, China) and reverse-transcribed into cDNA following the instructions of the PrimeScript RT Reagent Kit (Takara, Tokyo, Japan). The relative quantification of gene expression was presented using qRT-PCR on a LightCycler 96 real-time PCR system (Roche, Switzerland) with specific primers (Supplementary Table 1). Two internal reference genes, including GAPDH1 and GAPDH2, were used to normalize the gene expression (Supplementary Table 1) and calculated using the comparative 2^–Δ^ ^Δ^ ^*CT*^ method (Livak and Schmittgen, 2000). Three independent biological replicates were performed for each testing group.

### GspInv Purification and Invertase Activity Assay

W5 mycelia cultured in liquid VI medium for 2 days were withdrawn. Its cytoplasm proteins were extracted by treating the ground mycelia for 10 min in 50 mM HEPES-NaOH (pH 7.5), 1 mM EDTA, 20% (v/v) glycerol, 20 mM β-mercaptoethanol, and 0.05% (v/v) protease inhibitor cocktail (Sigma-Aldrich, St. Louis, MO, United States) (Hu et al., 2019). The protein extracts were used for enzyme purification. In detail, the crude extracts were loaded onto a high Q support column (Bio-Rad) pre-equilibrated with 50 mM HEPES-NaOH (pH 6.5), 1 mM EDTA, 5 mM β-mercaptoethanol, and 20% (v/v) glycerol. The proteins were eluted with a 0–0.5 M NaCl linear gradient in the equilibration buffer. The fractions containing invertase activity were pooled, concentrated in a low-binding regenerated cellulose membrane, and further purified via gel filtration through Sephacryl S100 (Amersham Pharmacia) pre-equilibrated with 50 mM citrate–Na_2_HPO_4_ buffer (pH 6.5), 1 mM EDTA, 10% (v/v) glycerol, 5 mM β-mercaptoethanol, and 150 mM NaCl. The purified protein was identified using LC-ESI-MS/MS (LTQ, Thermo Fisher Scientific, Shanghai, China) and searching the data reported by Zhou et al. (2020). The homogeneity of the purified protein was determined using 15% native polyacrylamide gel electrophoresis (Native-PAGE). Then, the protein was incubated in citrate–Na_2_HPO_4_ buffer (50 mM, pH 5.0) containing 200 mM sucrose at 45°C for 30 min and actively stained using 100 mM of NaOH solution that contained 0.2% triphenyl tetrazolium chloride after the removal of sucrose solution (van Wyk et al., 2013).

GspInv activity was routinely assayed with 50 mM sucrose as the substrate at 40–65°C. The optimal pH of GspInv was determined using citrate–Na_2_HPO_4_ buffer (pH 3.5–7.0) at optimal temperature. The kinetic constants of GspInv were determined in the presence of 1–50 mM sucrose, cellobiose, trehalose, maltose, isomaltose, raffinose, melibiose, and stachyose at optimal temperature and pH. The products of GspInv activity were quantified using HPLC equipped with a TSKgel Amide-80 column (4.6 mm × 250 mm, 5 μm, Tosoh Corporation, Kyoto, Japan) and an evaporative light-scattering detector 2424 (Waters, United States). The eluting solution was composed of acetonitrile and water (70:30, v/v).

### Expression of *GspInv*, *GspSUT1*, and *MtSWEET15.3* in *S. cerevisiae*

*Gongronella* sp. w5 was purely cultured for 24 h using sucrose as the carbon source, as previously described by Pan et al. (2014). The mycelia were then harvested and ground with a mortar and pestle in the presence of liquid nitrogen. Total RNA and cDNA were obtained as described above. The full-length *GspInv* cDNA was amplified using a primer pair of *GspInv*-f1 and *GspInv*-r1 (Supplementary Table 1). The amplified fragment was digested with *Xho*I and *Sac*II and ligated into vector pDR195 digested with the same enzymes. The constructed plasmid pDR195:*GspInv* was transformed into invertase-deficient yeast *S. cerevisiae* SEY2102. Transformants were selected on uracil-deficient synthetic complete (SC) medium with glucose (2%) at 30°C. After *GspInv* was confirmed in the transformants via PCR analysis, the positive transformants were cultured on uracil-deficient SC medium with sucrose (2%) as the sole carbon source. The transformants with empty vector pDR195 were used as the negative controls (Chang et al., 2017).

The plasmids pDR195:*GspSUT1*, pPMA1:*GspSUT1*, and pPMA1:*MtSWEET15.3* were constructed in the same way as pDR195:*GspInv* using primers pDR-GspSUT1-f and pDR-GspSUT1-r, Pma-GspSUT1-f and Pma-GspSUT1-r, and Pma-MtSWEET15.3-f and Pma-MtSWEET15.3-r, respectively (Supplementary Table 1). The plasmids were transformed into the invertase-deficient yeast *S. cerevisiae* SEY2102 (Chang et al., 2017) or the MST mutant yeast *S. cerevisiae* EBY. VW4000 (Wieczorke et al., 1999) as required.

The transformants were serially diluted (1-, 5-, 25-, and 125-fold) and cultured on uracil-deficient synthetic dropout SD medium, with 2% and 4% (m/v) maltose (as the control), sucrose, fructose, or glucose as the carbon source. The abilities of GspSUT1 and MtSWEET15.3 to transport sucrose were determined using yeast strains transformed with empty vectors, pDR195:*GspSUT1*, pPMA1:*GspSUT1*, and pPMA1:*MtSWEET15.3*, and ^3^H-sucrose according to the method of Zhou et al. (2007) and Wahl et al. (2010). Briefly, yeast cells of each transporter clone were grown in SD medium to an *OD*_600_ of 0.6–0.8, harvested by centrifugation (3000 × *g*, 4°C, 5 min), washed twice with 25 mM PBS buffer (pH 5.5), and resuspended in PBS buffer to a final *OD*_600_ of 20.0. Cells were shaken in a rotary shaker at 30°C, and transport tests were started by adding labeled ^3^H-Sucrose (0.5 μCi). Yeast cells were then harvested after 4-min incubation, filtered on microfiber filters under vacuum, and washed rapidly three times with 4 ml of ice-cold water. The incorporation of radioactivity was determined by scintillation counting. Total counts (CPM) were used to show the absorption of sucrose by GspSUT1 and MtSWEET15.3.

### Sequence Analysis

Sequence similarity search of GspInv and GspSUTs was performed using BlastP at NCBI^7^. The sequences that shared the highest sequence similarities with GspInv and GspSUTs in the GenBank RefSeq database and Glomeromycotina and Mucoromycotina genomes were chosen to construct the phylogenetic tree. Multiple sequence alignment of GspInv with related invertase sequences was performed using Clustal Omega^8^ (Sievers et al., 2011) and GeneDoc (Version 2.6.0.2) (Nicholas and Nicholas, 1997). The phylogenetic trees were constructed using the MEGA 7 program (Kumar et al., 2016) based on the maximum likelihood method with 1000 bootstrap replicates. SignalP5^9^ was to predict the putative signal peptide (Armenteros et al., 2019).

The genome sequences of 23 species, including seven Glomeromycotina, 15 Mucoromycotina, and *Rozella* species (selected as the outgroup) that could be used in the GenBank database, were employed for bioinformatic analysis. The protein sequences of single-copy genes from 23 species were extracted via OrthoMCL (version 2.0.9) (Li et al., 2003). The phylogenetic tree was constructed using raxmlHPC software based on the maximum likelihood method. R8S v.1.8 software (Sanderson, 2003) with a local molecular clock and three possible calibration points was used to date the divergence times in the tree, with the most recent common ancestors of *Rozella* constrained to 750 Ma (Chang et al., 2015), Glomeromycotina to 407 Ma, and *Phycomyces blakesleeanus* and *Lichtheimia corymbifera* to 200–300 Ma (Chang et al., 2019).

### Statistical Analysis

The statistical analyses in this study were carried out by Statistical Program SPSS 22.0 for Windows (SPSS Inc., Chicago, IL, United States). The mean values of the samples of different cultivations were compared by ANOVA tests. Duncan’s multiple range test was used for means separation. The level of significance was set at *p* < 0.05.

## Results

### W5 Promoted *M. truncatula* Growth

*Medicago truncatula* was used as the host plant to facilitate in-depth investigations on the effect of w5 on plant growth because of its fast growth characteristic. According to our protocol (Figure 1A), w5 and *M. truncatula* started to interact on agar plates from the 6th day. After about 20 days of cultivation, leaves of *M. truncatula* turned yellow and declined due to space and nutrition limitations. As a result, the effect of w5 on *M. truncatula* growth was tested at 16 dpi of w5. The fresh weight, dry weight, root diameter, total root length, root area, leaf area, and content of chlorophyll SPDA level of *M. truncatula* increased 0.5–1.0 times at 16 dpi of w5 (Table 1). The mycelia of w5 spread out of the root and throughout the root cells of *M. truncatula* (Figures 1B,C), which was also observed during *A. chinensis*–w5 interaction (Dong et al., 2018). The infection rate of w5 on *M. truncatula* root was 80.22 ± 3.11% as counted and calculated from 60 root segments. W5 spores were also observed inside the root hairs (Figure 1D).

**TABLE 1:** Effect of w5 on *M. truncatula* growth.

**Groups**	**Root diameter (mm)**	**Root length (cm)**	**Area of leaves (cm^2^)**	**Area of roots (cm^2^)**	**Fresh weight (g)**	**Dry weight (g)**	**Chlorophyll SPAD value**
− w5	0.440.03	9.710.90	4.842.69	1.340.05	0.120.01	0.0140.013	22.991.20
+ w5	0.670.11**	16.481.72**	7.331.38	2.450.34**	0.200.03*	0.0290.001**	33.392.19**

*P < 0.05; **P < 0.01.

### Global Changes in Metabolites and Transcriptome in *M. truncatula*

The metabonomic profiles of *M. truncatula* root tissues with or without w5 inoculation were analyzed and compared to explore the biocommunication between w5 and *M. truncatula*. A total of 72 metabolic compounds were significantly accumulated after w5 inoculation, including (iso)flavonoid (fold change of 1.79–2.86), phenylpropanoids, and hormones [salicylic acid (SA)] involved in plant defense reactions. Carbohydrates, such as 16-hydroxypalmitic acid (fold change of 3.1), D-Tagatose (fold change of 1.94), sucrose (fold change of 1.81), and D-Mannose (fold change of 1.76), were also accumulated in root tissues (Supplementary Appendix 2). These compounds are involved in a range of biological functions, including isoflavonoid biosynthesis, galactose metabolism, metabolic pathways, biosynthesis of phenylpropanoids, and starch and sucrose metabolism (Supplementary Figure 2A and Supplementary Appendix 1, 2).

Transcriptomic analysis showed that 790 genes were differentially expressed (log_2_ fold change >1) in *M. truncatula* roots by w5 inoculation. Out of these genes, 350 were upregulated and 440 were downregulated (Supplementary Appendix 3). RNA-Seq data were submitted to the Gene Expression Omnibus database (GEO accession GSE155951). qRT-PCR analysis using five randomly selected genes validated the reliability of the transcriptome data (Supplementary Table 2). PCA revealed that the observed variability differences between *M. truncatula* roots with or without w5 inoculation were the significant variance among the samples (Supplementary Figure 1). The transcriptomic and metabonomic results were mutually supportive according to association analysis (Supplementary Figure 3). Based on transcriptomic data, the genes related to photosynthesis, flavonoid and isoflavone biosynthesis, carbohydrate metabolism, cell wall, and lipid biosynthesis were significantly enriched (Supplementary Figures 2–5), revealing that w5 triggered the transcriptional and metabolic reprogramming of root cells and leading to a redistribution of metabolites involved in carbon metabolism.

### Transcription of Genes Related to Sucrose Metabolism in *M. truncatula* During Interaction

**FIGURE 2:**
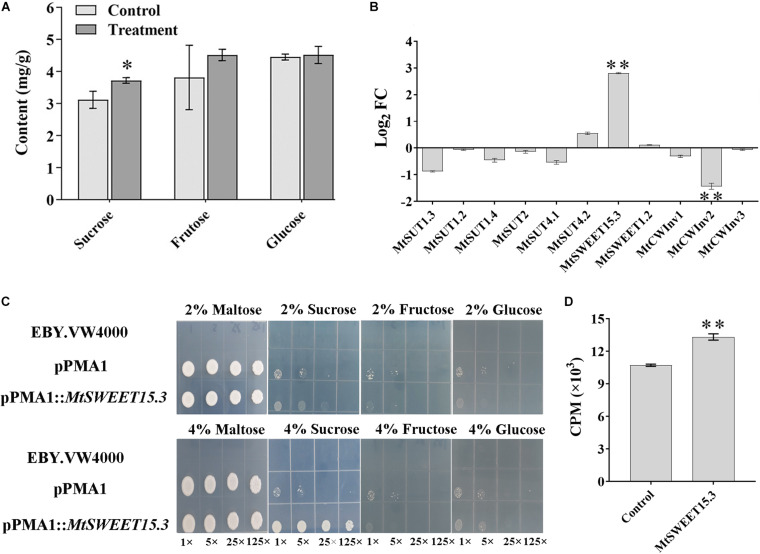
Changes of sugar and related genes in *M. truncatula* roots after w5 inoculation. **(A)** Changes in carbohydrates. **(B)** Fold changes (FC) of *M. truncatula* sucrose transporter (SUT) genes, SWEET genes, and cell-wall invertase (CWInv) genes at 16 dpi. **(C)** Complementation of the yeast hexose transport-defective strain EBY.VW4000 with MtSWEET15.3. The yeast cells were grown on SD media containing different sugars as sole carbon source at 30°C for 3 days. In each image, the yeast strain was diluted to an *OD*_600_ of 0.2 and then ×1, ×5, ×25, and ×125 diluted with sterile water. **(D)** Sucrose uptake experiment using ^3^H-sucrose as the substrate. *p < 0.05; **p < 0.01.

The synthesis, transport, storage, and degradation of the photosynthetic product, which is mainly sucrose, is one of the demanding factors in managing biomass allocation, plant productivity, and plant interactions with various soil microbes. Given that sucrose concentration and genes related to carbohydrate metabolism were significantly accumulated in the root tissue after w5 inoculation, the sucrose, fructose, and glucose contents in *M. truncatula* roots were quantitatively analyzed. After w5 treatment, the sucrose content in *M. truncatula* roots was significantly higher than that in the control group (Figure 2A). Fructose also tends to accumulate in roots after inoculation with w5. By contrast, w5 inoculation did not affect the glucose content in root tissues, which may be caused by the exportation of glucose into w5 by some root-driven SWEET transporters (Figure 2A).

The accumulation of sucrose in root tissue triggered us to explore the possibility of transporting sucrose from *M. truncatula* to w5. To address this question, sucrose metabolism-related genes, including CWInv, SWEETs, and SUTs, were specifically analyzed based on the transcriptome data. Except for MtCWInv2 (Medtr1g015980), which was slightly downregulated in the transcriptome, the other six *CWInv*s in *M. truncatula* remained unchanged. Among the 26 SWEET genes (An et al., 2019), *MtSWEET15.3* (Medtr7g405730, log_2_ fold change of 3.28) was the only gene upregulated, with others remaining unchanged during plant–w5 interaction. A total of six SUTs were predicted in the *M. truncatula* genome. They remained unchanged during the interaction. These results suggested that sucrose or its hydrolysates may be transported from *M. truncatula* to w5 via *MtSWEET15.3*. Results from qRT-PCR analysis using the root samples collected at 16 dpi further validated this prediction. Similar to the transcriptome results, none of the six SUTs in *M. truncatula* was significantly upregulated. The SWEET gene *MtSWEET15.3* was 2.81 times upregulated. Moreover, two CWInv genes remained basically unchanged (log_2_ fold change <1) after w5 inoculation, with the CWInv gene MtCWInv2 (log_2_ fold change <−1) downregulated among the three CWInv genes in *M. truncatula* root cells (Figure 2B).

Yeast mutant complementation experiments were conducted to test the ability of MtSWEET15.3 to transport sugars. Results showed that *S. cerevisiae* EBY.VW4000 expressing MtSWEET15.3 can help yeast cells grow on 4% sucrose, but not on 4% glucose and 4% fructose. By comparison, yeast cells grew inactively and showed no apparent difference in growth under 2% sugars (Figure 2C). Sucrose uptake was analyzed with radiolabeled ^3^H-sucrose as the substrate. Results also showed that MtSWEET15.3 possessed the ability to transport sucrose into the cytoplasm (Figure 2D). In conclusion, all these results suggested that together with its accumulation in roots, sucrose rather than glucose and fructose was accelerated to be transported out of root cells by SWEETs such as MtSWEET15.3 because CWInvs, which were utilized by plants to hydrolyze sucrose into glucose and fructose, were not upregulated during the interaction.

### GspInv and GspSUT1 Are Proteins Involved in Sucrose Metabolism in w5

**FIGURE 3:**
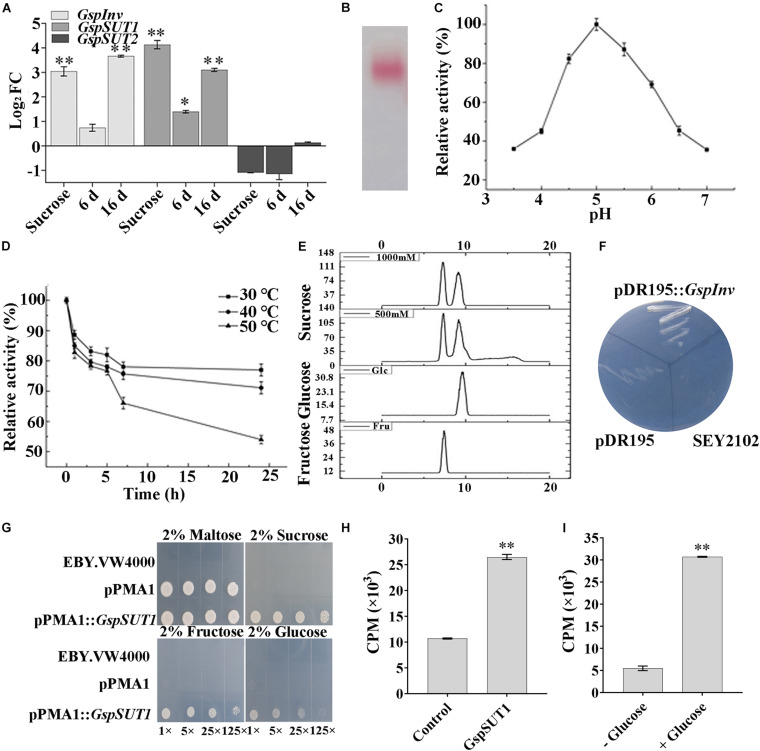
Activity and function determination of GspInv and GspSUT1.**(A)** Fold changes of w5 invertase gene (*GspInv*) and sucrose transporter genes (*GsSUT1* and *GsSUT2*) after 24 h cultivation in the presence of sucrose or after co-cultivated with *M. truncatula* at 6 dpi and 16 dpi. The control group was w5 cultured on medium without carbon source. Values are the means ± standard (*n* = 9). **(B)** Native-PAGE of purified GspInv. **(C,D)** Effects of pH and temperature on activity of GspInv. **(E)** Analysis of sugar products using 1000 mM and 500 mM sucrose as the substrate to test transglycosidase activity. **(F)** Heterologous expression of GspInv in invertase-deficient yeast *S. cerevisiae* strain SEY2102 on synthetic medium with sucrose as the sole carbon source. **(G)** Complementation of the yeast hexose transport-defective strain EBY.VW4000 with GspSUT1. The yeast cells were grown on SD media containing different sugars as sole carbon source at 30°C for 3 days. In each image, the yeast strain was diluted to an *OD*_600_ of 0.2 and then ×1, ×5, ×25, and ×125 diluted with sterile water. **(H)** Sucrose uptake experiment using ^3^H-sucrose as the substrate. **(I)** GspSUT1 is activated in the presence of the metabolizable carbon source glucose in SEY2102. *p < 0.05; **p < 0.01.

Results mentioned above indicated that w5 might directly uptake sucrose released from *M. truncatula* root cells as the carbon source for growth. Because an invertase (*GspInv*) and two SUT genes (*GspSUT*s) were previously predicted in the w5 genome (Hu et al., 2019), the transcription levels of the *GspSUT*s and *GspInv* of w5 were monitored using qRT-PCR during w5–plant interaction to address the prediction mentioned above. The *GspInv* transcription level remained unchanged compared with the control groups at 6 dpi but highly upregulated at 16 dpi (Figure 3A). After the co-cultivation of w5 with plants, the expression of *GspSUT1* was unchanged at 6 dpi but upregulated at 16 dpi (Figure 3A). On the contrary, the expression of *GspSUT2* was always downregulated at 6 dpi and unchanged at 16 dpi (Figure 3A). The *GspInv*, *GspSUT1*, and *GspSUT2* transcription levels were also analyzed by culturing w5 in liquid cultures using sucrose as the sole carbon source. Interestingly, the *GspInv* and GspSUT1 transcription levels largely increased when using sucrose as the carbon source, whereas the *GspSUT2* transcription level was unchanged throughout the experiment (Figure 3A). These results suggested that *GspInv* and *GspSUT1* may be involved in sucrose metabolism during the interaction between w5 and *M. truncatula*.

The invertases from the GH32 family are essential for fungi to use sucrose as the carbon source (Parrent et al., 2009; Doidy et al., 2012). The invertase activity was previously detected from the cytoplasmic extracts of w5 when cultured using sucrose as the carbon source (Hu et al., 2019). The protein with invertase activity was thus purified from the w5 mycelia collected after 48 h cultivation, with sucrose as the carbon source, to provide an improved understanding of invertase in w5. After the sequential purification steps were performed, MALDI–TOF–MS/MS identification of the purified protein with pink color in Native-PAGE gel showed that the target protein shared the same peptide sequence with rGspInv that was heterologously expressed in *Komagataella pastoris* (Zhou et al., 2020; Figure 3B). The purified GspInv also displayed biochemical properties similar to those of rGspInv (Zhou et al., 2020), with an optimal pH of 5.0 for activity (Figure 3C). GspInv was stable at 30°C and 40°C, with 70% of the original activity retained after 24 h of incubation at 40°C (Figure 3D). The substrate affinity of GspInv toward sucrose was 3.1 mM, with glucose and fructose as the end products. No transglycosidase activity was detected in GspInv (Figure 3E). Different from rGspInv, which showed similar specific activities toward sucrose and raffinose and no activity to stachyose (Zhou et al., 2020), GspInv hydrolyzed sucrose, raffinose, and stachyose, with specific activities of 3380 ± 26, 208 ± 14, and 128 ± 10 U/mg, respectively (Table 2). GspInv also showed no activity to trehalose, cellobiose, maltose, melibiose, and isomaltose. These results confirmed that w5 has an intracellular acid GspInv with biochemical characteristics similar to those of most characterized fungal acidic invertases activated under sucrose or plant root induction.

**TABLE 2:** Substrate specificity of GspInv.

**(C_6_H_10_O_5_)n**	**Substrate**	**Linkage**	**Specific activity (U mg^–1^)**
*N* = 2	Sucrose	O-α-D-glucopyranosyl-(1-2)-β- D-fructopyranoside	3380 ± 26
	Trehalose	O-α-D- glucopyranosyl -(1-1)-α- D- glucopyranoside	0
	Cellobiose	O-β-D- glucopyranosyl -(1-4)- D- glucopyranoside	0
	Maltose	O-α-D- glucopyranosyl -(1-4)- D- glucopyranoside	0
	Isomaltose	O-α-D- glucopyranosyl -(1-6)- D- glucopyranoside	0
*N* = 3	Raffinose	O-α-D- galactopyranosyl -(1-6)-D- glucopyranosyl -(1-2)-β-D- fructopyranoside	208 ± 14
	Melizitose	O-α-D- glucopyranosyl -(1-6)-D- fructofuranosyl -(1-4)-D- glucopyranoside	0
*N* = 4	Stachyose	O-α-D-fructofuranosyl-(1-6)-D-galactopyranosyl-(1-6)- D-galactopyranosyl-(1-6) -D-glucopyranoside	128 ± 10
*N* > 20	Inulin	O-β-D-fructofuranosyl-(2-1)-[D-galactopyranosyl]n–D-glucopyranoside	0

The complete GspInv cDNA was cloned into the expression vector pDR195 and transformed into *S. cerevisiae* deficient in invertase gene to verify that GspInv was invertase *in vivo*. The pDR195:*GspInv* transformants could complement the invertase-negative phenotype of this strain and grow on the medium with sucrose as the sole carbon source (Figure 3F). By contrast, the pDR195 transformants could not succeed, although both types of transformants showed no difference in growth on SC medium with glucose as the carbon source (data not shown). These results further confirmed that GspInv acted as invertase *in vivo*.

Using the same strategy, *GspSUT1* was transformed into the MST mutant yeast *S. cerevisiae* EBY.VW4000 to verify its function. Yeast cells transformed with pPMA1 and pPMA1:*GspSUT1* could grow when using maltose as the carbon source and showed no apparent difference in growth. By comparison, only pPMA1:*GspSUT1* transformants could grow on the medium with sucrose, glucose, or fructose as the sole carbon source. pPMA1:*GspSUT1* transformants prefer sucrose rather than glucose and fructose, as shown in Figure 3G. The transport capacity of GspSUT1 to sucrose was tested using ^3^H-sucrose. The CPM value of pPMA1:*GspSUT1* transformants was three times higher than that of the yeast transformed with pPMA1 (Figure 3H). Because the *S. cerevisiae* strain EBY.VW4000 encodes an extracellular invertase that slowly hydrolyzes extracellular sucrose, the invertase-deficient yeast strain *S. cerevisiae* SEY2102 expressing GspSUT1 was also constructed and tested in sucrose uptake capacity. Sucrose was expected to be imported into the cell when GspSUT1 is expressed but cannot be hydrolyzed due to a lack of invertase activity (Emr et al., 1983). In the presence of glucose, which was recognized as the primary carbon source for *S. cerevisiae* SEY2102, sucrose uptake was strongly enhanced compared to that without glucose addition (Figure 3I). These results suggested that *GspSUT1* is a sugar transporter that prefers to transport sucrose than glucose and fructose.

### GspInv and GspSUT1 Represent Novel Sucrose-Related Proteins in Plant Beneficial Fungi

**FIGURE 4:**
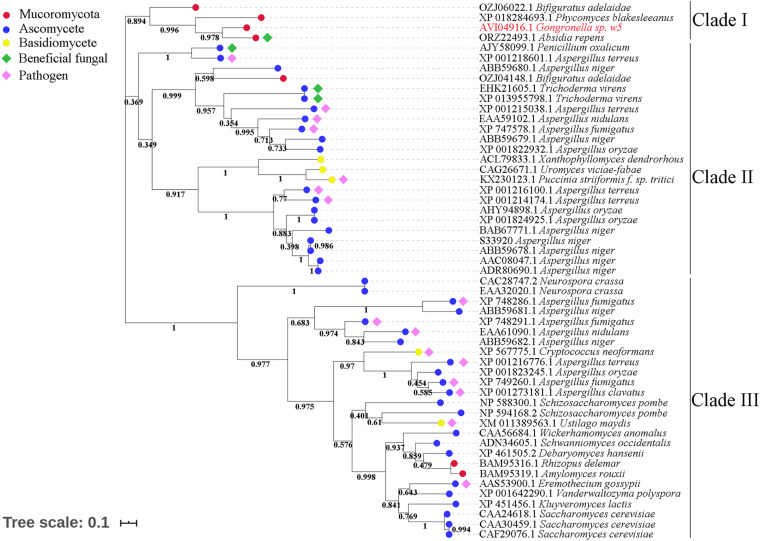
Phylogenetic tree of fungal invertases.

The invertases from the GH32 family are essential for fungi to use sucrose as the carbon source (Parrent et al., 2009; Doidy et al., 2012). Although GspInv shared very low sequence identities with the sequences in the GenBank database (Hu et al., 2019; Zhou et al., 2020), the results of sequence alignment conducted between GspInv and other known GH32 invertases showed that they shared the same conserved motifs (Supplementary Figure 6; Zhou et al., 2020), thereby supporting the conclusion that GspInv is a member of the GH32 family (Parrent et al., 2009). After clustering GspInv with 52 fungal-origin invertases that shared the highest sequence similarities with GspInv in the NCBI RefSeq database, a phylogenetic tree that contained three clades were obtained (Figure 4). Five of seven invertases from Mucoromycota, including GspInv, were branched into the clade I and located near invertases from beneficial fungi, including *T. virens* (GenBank Nos. XP_013955798 and EHK21605) and *Penicillium oxalicum* (AJY58099).

**FIGURE 5:**
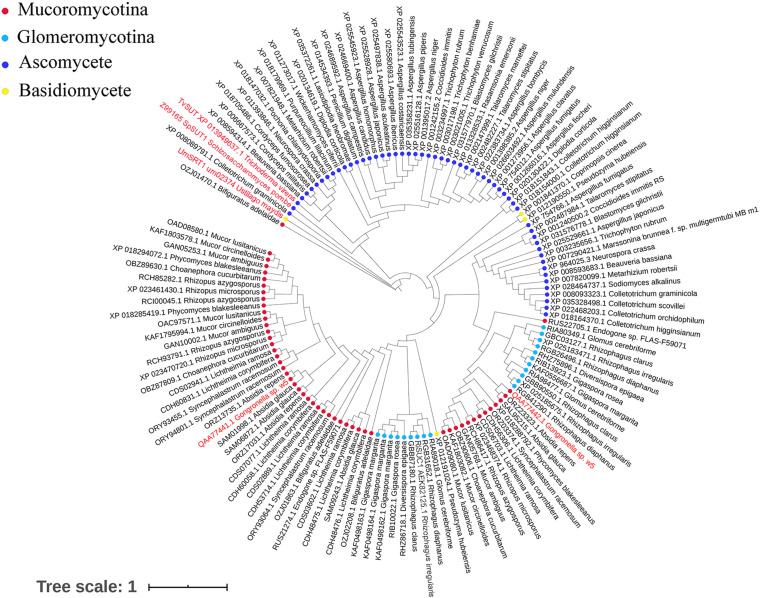
Phylogenetic tree of fungal sucrose transporters. The red characters are SUTs from w5 and SUTs of fungus, which have been verified.

GspSUT1 and GspSUT2 amino acid sequences were also compared with the reported fungal SUTs. The two sequences shared very few conserved domains with the three characterized fungal SUTs (Supplementary Figure 7). This phenomenon may be explained by the low sequence identities between GspInv and other sequences (28%) and *T. virens* proteins (32%). The results derived from the constructed phylogenetic tree, which contained 131 fungal SUTs, showed that GspSUT1 and GspSUT2 were clustered on two different clusters and far away from three characterized SUTs, including SpSUT1 from *Schizosaccharomyces pombe* (O14091.1) (Reinders and Ward, 2001; Sun et al., 2010), TvSUT from *T. virens* (XP_013949637.1) (Vargas et al., 2011), and UmSRT1 from *Ustilago maydis* (UM02374) (Wahl et al., 2010; Figure 5). SUTs from Mucoromycotina and Glomeromycotina were clustered together, possibly indicating that they derived from a single ancestral gene. All these results indicated that GspSUT1 is a novel SUT.

### Phylogenetic Placement and Dating of Mucoromycotina and Glomeromycotina

**FIGURE 6:**
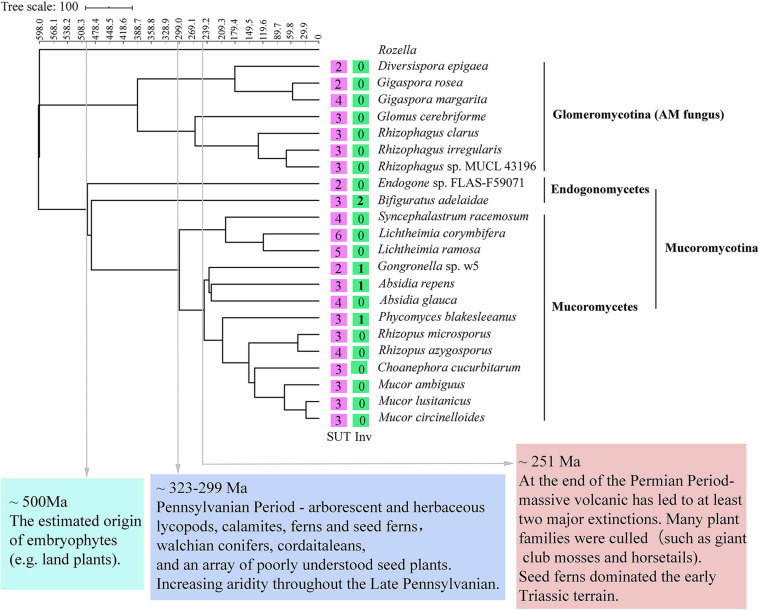
Phylogenetic placement and divergence time estimation of Mucoromycotina and Glomeromycotina. The numbers in the box represent the number of sucrose transporters (SUTs) and invertases (Invs).

A species tree was constructed to obtain a deep insight into the evolutionary history of invertases and SUTs among Glomeromycotina, Endogonomycetes, and Mucoromycetes (Figure 6). The beneficial fungal species with genome sequence in the GenBank database, including seven from Glomeromycotina, two from Endogonomycetes, and 13 from Mucoromycetes, were selected. The reconstructed relationships in the maximum likelihood tree from this study were consistent with those reported previously by Chang et al. (2019), suggesting its reliability. Following the phenomenon that AM fungi could not assimilate sucrose directly, none of the seven Glomeromycotina contained putative invertases, although more than two putative SUTs were predicted in their genomes (Supplementary Appendix 4). The results from the tree also suggested “retain and loss” of the sucrose utilization ability of Mucoromycetes during evolution. Based on BLAST results, all the 15 Mucoromycotina strains contained variable SUTs in their genomes. However, only four of them had invertases, including *Bifiguratus adelaidae* (two), w5 (one), *Absidia repens* (one), and *P. blakesleeanus* (one), which were all predicted to exist at approximately 500, 300, 230, and 200 Ma ago. Other species lost invertase genes completely during evolution.

## Discussion

Mucoromycotina-like fungi are recognized as one of the earliest fungi to form symbiotic relationships with plants in the ancient land (Field et al., 2015, 2019; Martin et al., 2017). They establish mutualistic relationships with many extant non-vascular plants, such as liverworts and hornworts, and some early diverging vascular plants (Hoysted et al., 2018). However, research on the interaction between Mucoromycotina and higher plants is still in its infancy (Hoysted et al., 2018). Studies on the interactions of Mucoromycotina with plants and their utilization of plant carbon sources could help further understand the evolutionary history of early terrestrial plants and symbiotic fungi. This study presented evidence that suggested a novel relationship between *Gongronella* sp. w5, which is a Mucoromycotina reported to be widely spread over the world (Rodrigues et al., 2014; Li et al., 2016; You et al., 2017; Cruz-Lachica et al., 2018; Mandl et al., 2018), and *M. truncatula*, which is a model plant that can form symbiotic relationships with various AM fungi.

Sucrose, which is produced in the source leaves and transported in heterotrophic tissues, is one of the primary photosynthetic products controlling carbohydrate distribution and signaling in plant–fungi interactions. During a *Trichoderma harzianum*–tomato interaction, the beneficial fungus *T. harzianum* promotes tomato growth by increasing sucrose in the root (Fiorini et al., 2016). The plant-derived sucrose is a key element in the symbiotic association between maize plants and the beneficial fungus *T. virens* (Vargas et al., 2009). The present study also showed that w5 inoculation led to changes in sucrose metabolism pathways and the enhancement of sucrose storage in *M. truncatula* sink roots (Figure 2 and Supplementary Figure 2). Metabolomic and transcriptomic results demonstrated that the starch in the root also accumulated (Supplementary Figure 5). Given that the chlorophyll content, leaf area, and fresh weight significantly increased during w5 inoculation (Table 1), we concluded that w5 invasion changed the relationship between source and sink, increased photosynthesis and the movement of carbohydrates from leaves to roots, and finally resulted in plant growth and sucrose accumulation in root tissues (Vargas et al., 2009; Fiorini et al., 2016).

In mycorrhizal associations, mycorrhizal fungi could not directly utilize sucrose and must rely on plant CWInvs to cleavage sucrose into equimolar concentrations of glucose and fructose to support the growth of fungal cells (Ruan, 2014; Roth and Paszkowski, 2017; Wipf et al., 2019). None of the six CWInvs of *M. truncatula* was upregulated after w5 inoculation, suggesting that w5 may not need to rely on CWInvs to hydrolyze sucrose into glucose to survive. The fact that w5 grows well in liquid cultures with sucrose than with glucose or fructose as the sole carbon source reinforced this conclusion (Hu et al., 2019). In addition, one of the SWEET genes (*MtSWEET15.3*) was upregulated in *M. truncatula* during w5–plant interaction. MtSWEET15.3 belongs to clade III of the plant SWEET family. Results from the yeast function complementation experiment and sucrose uptake experiment in this study showed that MtSWEET15.3 possesses the ability to transport the sucrose into the cell (Figure 2). Results from other groups also evidenced that SWEET15 members mediate sucrose transport but not glucose in cells such as *Xenopus oocytes* (Wang et al., 2019) and HEK293T (Chen et al., 2012). All the results suggested that the transport of sucrose rather than glucose and fructose by *M. truncatula* root cells may be accelerated.

Simultaneously, a novel cytoplasmic acid invertase GspInv and a SUT GspSUT1 were upregulated in w5 during its interaction with *M. truncatula* or in cultures with sucrose as the carbon source (Figures 3–5). GspInv and GspSUT1 functioned as invertase and SUT *in vivo*, respectively. These results suggest that after it was exported by *M. truncatula*, sucrose is likely to be direct uptake by w5 from *M. truncatula* using GspSUT. Then, the sucrose was taken up into the cell and hydrolyzed in the cytoplasm by GspInv to support mycelial development. Thus, this study reported that a plant beneficial fungus from Mucoromycete has the ability to directly uptake sucrose from plant cells during their interaction. This study also provides novel insights into carbohydrate metabolism and the role of invertase during fungus–plant interaction.

Several works have reported the direct usage of plant sucrose by fungi (Vargas et al., 2009, 2011; Wahl et al., 2010; Chang et al., 2017) and some bacteria (Parrent et al., 2009). Evidence has shown that the direct absorption of sucrose is one of the strategies adopted by pathogenic fungi to avoid triggering the host plant defenses (Wahl et al., 2010; Chang et al., 2017). By comparison, in a beneficial fungus–plant relationship, sucrose degradation in fungal cells may be considered an extension of plant carbohydrate metabolism that increases the demand for sugars and systemically alters plant metabolism (Vargas et al., 2009). During the process, the carbohydrate flow to the symbiotic partner is controlled by the plant through sucrose hydrolysis (Ruan, 2014). Plants could detect, discriminate, and reward the best fungal partners with more carbohydrates, suggesting that the symbiont also could not be “enslaved” between a plant and beneficial fungus symbionts (Kiers and Denison, 2008). Unfortunately, due to the difficulties in knocking out the sucrose metabolism-related genes in w5, the exact mechanisms controlling sucrose metabolism in w5 need further investigation.

The symbiotic relationship of AM fungi from Glomeromycotina with early diverging liverworts must be maintained by selection pressures, leading to the loss of invertases or sucrose synthase genes for sucrose hydrolysis (Wipf et al., 2019). The lack of invertases in seven Glomeromycotina species demonstrated in this study was consistent with the previous reports (Wipf et al., 2019). Although putative SUTs were identified in Glomeromycotina, their role remains unknown (Helber et al., 2011; Wipf et al., 2019). Selection pressures make the symbiotic relationship between Mucoromycotina and plants undergo a process of “loss and gain” (Rimington et al., 2019). They seem not to affect Mucoromycotina SUT genes but indeed on their invertases (Figure 6). Besides, invertases exist in several Mucoromycotina species that occupy the important time nodes related to essential issues in the evolution history on the maximum likelihood tree. For example, *B. adelaidae*, a member of Endogonomycetes, was the first fungus to evolve with two invertase genes, dating to approximately 500 Ma, which is also the time of origin of embryophytes (e.g., land plants) (Chang et al., 2019; Figure 6). Species with invertase genes, including w5, *A. repens*, and *P. blakesleeanus*, were all predicted to exist at approximately 300 to 200 Ma ago. Other species lost invertase genes completely during evolution. During this period, land plants gradually became abundant and seed plants emerged at approximately 323–299 Ma (Looy and Hotton, 2014) and massive volcanic eruptions led to at least two major extinctions and catastrophic diversity loss at approximately 250 Ma (Knoll and Nowak, 2017). Moreover, high CO_2_ concentration appeared to benefit the Mucoromycotina–plant partnerships (Field et al., 2015). In fact, fourfold increases in atmospheric CO_2_ levels occurred at the Triassic–Jurassic period (approximately 200 Ma) (Mander et al., 2010). Based on these facts, this study hypothesized that Mucoromycotina was able to utilize plant sucrose in their earliest evolution, with many species gradually losing and gaining invertase genes because of some reasons, such as mass extinctions and environmental changes.

In conclusion, our results showed that w5 could promote *M. truncatula* to grow better and accumulate sucrose in root tissue. W5 may communicate with the host plant by employing a novel GH32 invertase and a novel SUT (Supplementary Figure 8).

## Data Availability Statement

The datasets presented in this study can be found in online repositories. The names of the repository/repositories and accession number(s) can be found in the article/ Supplementary Material.

## Author Contributions

ZF and YX conceived and supervised the study. ZF and WF designed the experiments. XW, JF, and JL performed the experiments. XW, ZF, and PL analyzed the results. XW, PL, ZF, and YX wrote the manuscript. All authors reviewed the manuscript.

## Conflict of Interest

The authors declare that the research was conducted in the absence of any commercial or financial relationships that could be construed as a potential conflict of interest.

## Footnotes

1 http://www.kegg.jp/

2 www.omicsbean.cn

3 http://www.medicagogenome.org/

4 http://www.ncbi.nlm.nih.gov/

5 http://www.expasy.ch/sprot/

6 http://www.ncbi.nlm.nih.gov/COG/

7 http://www.blast.ncbi.nlm.nih.gov

8 https://www.ebi.ac.uk/Tools/msa/clustalo/

9 http://www.cbs.dtu.dk/services/SignalP/

## References

[B1] An J. Zeng T. Ji C. De Graaf S. Zheng Z. Xiao T. T. (2019). A Medicago truncatula SWEET transporter implicated in arbuscule maintenance during arbuscular mycorrhizal symbiosis. *New Phytol.* 224 396–408. 10.1111/nph.15975 31148173

[B2] Armenteros J. J. Tsirigos K. D. Sonderby C. K. Petersen T. N. Winther O. Brunak S. (2019). SignalP 5.0 improves signal peptide predictions using deep neural networks. *Nat. Biotechnol.* 37 420–423. 10.1038/s41587-019-0036-z 30778233

[B3] Behie S. W. Bidochka M. J. (2014). Nutrient transfer in plant–fungal symbioses. *Trends Plant Sci.* 19 734–740. 10.1016/j.tplants.2014.06.007 25022353

[B4] Benton H. P. Ivanisevic J. Mahieu N. G. Kurczy M. E. Johnson C. H. Franco L. (2015). Autonomous metabolomics for rapid metabolite identification in global profiling. *Anal. Chem.* 87 884–891. 10.1021/ac5025649 25496351 PMC4303330

[B5] Berbee M. L. James T. Y. Strulluderrien C. (2017). Early diverging fungi: diversity and impact at the dawn of terrestrial life. *Annu. Rev. Microbiol.* 71 41–60. 10.1146/annurev-micro-030117-020324 28525299

[B6] Bidartondo M. I. Read D. Trappe J. M. Merckx V. S. Ligrone R. (2011). The dawn of symbiosis between plants and fungi. *Bio Lett.* 7 574–577. 10.1098/rsbl.2010.1203 21389014 PMC3130224

[B7] Blee K. A. Anderson A. J. (2002). Transcripts for genes encoding soluble acid invertase and sucrose synthase accumu late in root tip and cortical cells containing mycorrhizal arbuscules. *Plant Mol. Biol.* 5 197–211. 10.1023/A:1016038010393 12175013

[B8] Bonfante P. Venice F. (2020). Mucoromycota: going to the roots of plant-interacting fungi. *Fungal Biol. Rev.* 34 100–113. 10.1016/j.fbr.2019.12.003

[B9] Chang Q. Liu J. Lin X. Hu S. Yang Y. Li D. (2017). A unique invertase is important for sugar absorption of an obligate biotrophic pathogen during infection. *New Phytol.* 215 1548–1561. 10.1111/nph.14666 28744865

[B10] Chang Y. Desiro A. Na H. Sandor L. Lipzen A. Clum A. (2019). Phylogenomics of *Endogonaceae* and evolution of mycorrhizas within *Mucoromycota*. *New Phytol.* 222 511–525. 10.1111/nph.15613 30485448

[B11] Chang Y. Wang S. Sekimoto S. Aerts A. Choi C. Clum A. (2015). Phylogenomic analyses indicate that early fungi evolved digesting cell walls of algal ancestors of land plants. *Genome Biol. Evol.* 7 1590–1601. 10.1093/gbe/evv090 25977457 PMC4494064

[B12] Chen L. Q. Qu X. Q. Hou B. H. Sosso D. Osorio S. Fernie A. R. (2012). Sucrose efflux mediated by SWEET proteins as a key step for phloem transport. *Science* 335 207–211. 10.1126/science.1213351 22157085

[B13] Cruz-Lachica I. Marquezzequera I. Allendemolar R. Sanudobarajas J. A. Leonfelix J. Leylopez N. (2018). Diversity of mucoralean fungi in soils of papaya (*Carica papaya* L.) producing regions in Mexico. *Fungal Biol. Rev.* 122 810–816. 10.1016/j.funbio.2018.04.008 30007431

[B14] Desiro A. Faccio A. Kaech A. Bidartondo M. I. Bonfante P. (2015). Endogone, one of the oldest plant-associated fungi, host unique *Mollicutes-related endobacteria*. *New Phytol.* 205 1464–1472. 10.1111/nph.13136 25345989

[B15] Desiro A. Rimington W. R. Jacob A. Pol N. V. Smith M. E. Trappe J. M. (2017). Multigene phylogeny of *Endogonales*, an early diverging lineage of fungi associated with plants. *IMA Fungus* 8 245–257. 10.5598/imafungus.2017.08.02.03 29242774 PMC5729711

[B16] Doidy J. Grace E. Kuhn C. Simonplas F. Casieri L. Wipf D. (2012). Sugar transporters in plants and in their interactions with fungi. *Trends Plant Sci.* 17 413–422. 10.1016/j.tplants.2012.03.009 22513109

[B17] Dong Y. Sun Q. Zhang Y. Wang X. Liu P. Xiao Y. (2018). Complete genome of *Gongronella sp*. w5 provides insight into its relationship with plant. *J. Biotechnol.* 286 1–4. 10.1016/j.jbiotec.2018.08.022 30194967

[B18] Emr S. D. Schekman R. Flessel M. C. Thorner J. (1983). An MF alpha 1-SUC2 (alpha-factor-invertase) gene fusion for study of protein localization and gene expression in yeast. *Proc. Natl. Acad. Sci. U S A.* 80 7080–7084. 10.1073/pnas.80.23.7080 6359161 PMC389996

[B19] Field K. J. Bidartondo M. I. Rimington W. R. Hoysted G. A. Beerling D. Cameron D. D. (2019). Functional complementarity of ancient plant–fungal mutualisms: contrasting nitrogen, phosphorus and carbon exchanges between *Mucoromycotina* and *Glomeromycotina* fungal symbionts of liverworts. *New Phytol.* 223 908–921. 10.1111/nph.15819 30919981

[B20] Field K. J. Cameron D. D. Leake, Tille S. Bidartondo M. I. Beerling D. J. (2012). Contrasting arbuscular mycorrhizal responses of vascular and non-vascular plants to a simulated *Palaeozoic* CO2 decline. *Nat. Commun.* 3:835. 10.1038/ncomms1831 22588297

[B21] Field K. J. Rimington W. R. Bidartondo M. I. Allison K. E. Beerling D. J. Cameron D. D. (2015). First evidence of mutualism between ancient plant lineages (Haplomitriopsida liverworts) and *Mucoromycotina* fungi and its response to simulated *Palaeozoic* changes inatmospheric CO2. *New Phytol.* 154 743–756. 10.1111/nph.13024 25230098 PMC4303992

[B22] Fiorini L. Guglielminetti L. Mariotti L. Curadi M. Picciarelli P. Scartazza A. (2016). Trichoderma harzianum T6776 modulates a complex metabolic network to stimulate tomato cv. *Micro-Tom growth. Plant Soil.* 400 351–366. 10.1007/s11104-015-2736-6

[B23] Gaude N. S. Bortfeld D. N. Lohse M. Krajinski F. (2012). Arbuscule containing and non-colonized cortical cells of mycorrhizal roots undergo extensive and specific reprogramming during arbuscular mycorrhizal development. *Plant J.* 69 510–528. 10.1111/j.1365-313X.2011.04810.x 21978245

[B24] Helber N. Wippel K. Sauer N. Schaarschmidt S. Hause B. Requena N. (2011). A versatile monosaccharide transporter that operates in the arbuscular mycorrhizal fungus *Glomus* sp is crucial for the symbiotic relationship with plants. *Plant Cell* 23 3812–3823. 10.1105/tpc.111.089813 21972259 PMC3229151

[B25] Hoffmann K. Pawlowska J. Walther G. Wrzosek M. De Hoog G. S. Benny G. L. (2013). The family structure of the *Mucorales*: a synoptic revision based on comprehensive multigene-genealogies. *Persoonia* 30 57–76. 10.3767/003158513X666259 24027347 PMC3734967

[B26] Hoysted G. A. Kowal J. Jacob A. S. Rimington W. R. Duckett J. G. Pressel S. (2018). A mycorrhizal revolution. *Curr. Opin. Plant Biol.* 44 1–6. 10.1016/j.pbi.2017.12.004 29289791

[B27] Hu J. Zhang Y. Xu Y. Sun Q. Liu J. Fang W. (2019). Gongronella sp. w5 elevates *Coprinopsis cinerea* laccase production by carbon source syntrophism and secondary metabolite induction. *Appl. Microbiol. Biotechnol.* 103 411–425. 10.1007/s00253-018-9469-4 30406450

[B28] Ivanisevic J. Zhu Z. Plate L. Tautenhahn R. Chen S. S. Obrien P. J. (2013). Toward ‘omic scale metabolite profiling: a dual separation–mass spectrometry approach for coverage of lipid and central carbon metabolism. *Anal. Chem.* 85 6876–6884. 10.1021/ac401140h 23781873 PMC3761963

[B29] James T. Y. Kauff F. Schoch C. L. Matheny P. B. Hofstetter V. Cox C. J. (2006). Reconstructing the early evolution of Fungi using a six-gene phylogeny. *Nature* 443 818–822. 10.1038/nature05110 17051209

[B30] Jiang Y. Wang W. Xie Q. Liu N. Liu L. Wang D. (2017). Plants transfer lipids to sustain colonization by mutualistic mycorrhizal and parasitic fungi. *Science* 356 1172–1175. 10.1126/science.aam9970 28596307

[B31] Kiers T. Denison R. F. (2008). Sanctions, cooperation, and the stability of plant-rhizosphere mutualisms. *Annu. Rev. Ecol. Evol. Syst.* 39 215–236. 10.1146/annurev.ecolsys.39.110707.173423

[B32] Knoll A. H. Nowak M. A. (2017). The timetable of evolution. *Sci. Adv.* 3:e1603076. 10.1126/sciadv.1603076 28560344 PMC5435417

[B33] Krishnakumar V. Kim M. Rosen B. D. Karamycheva S. Bidwell S. L. Tang H. (2015). MTGD: the medicago truncatula genome database. *Plant Cell Physiol.* 56 1123–1130. 10.1093/pcp/pcu179 25432968

[B34] Kumar S. Stecher G. Tamura K. (2016). MEGA7: molecular evolutionary genetics analysis version 7.0 for bigger datasets. *Mol. Biol. Evol.* 33 1870–1874. 10.1093/molbev/msw054 27004904 PMC8210823

[B35] Li L. Stoeckert C. J. Roos D. S. (2003). OrthoMCL: identification of ortholog groups for eukaryotic genomes. *Genome Res.* 13 2178–2189. 10.1101/gr.1224503 12952885 PMC403725

[B36] Li W. Wang M. Bian X. Guo J. Cai L. (2016). A high-level fungal diversity in the intertidal sediment of chinese seas presents the spatial variation of community composition. *Front. Microbiol.* 7:2098. 10.3389/fmicb.2016.02098 28066402 PMC5179519

[B37] Lindahl B. D. Tunlid A. (2015). Ectomycorrhizal fungi - potential organic matter decomposers, yet not saprotrophs. *New Phytol.* 205 1443–1447. 10.1111/nph.13201 25524234

[B38] Liu H. Hu C. Sun X. Tan Q. Nie Z. Hu X. (2010). Interactive effects of molybdenum and phosphorus fertilizers on photosynthetic characteristics of seedlings and grain yield of *Brassica napus*. *Plant Soil.* 326 345–353. 10.1007/s11104-009-0014-1

[B39] Livak K. J. Schmittgen T. D. (2000). Analysis of relative gene expression data using real-time quantitative PCR and the 2(-Delta C(T)) method. *Methods* 25 402–408. 10.1006/meth.2001.1262 11846609

[B40] Looy C. V. Hotton C. L. (2014). Spatiotemporal relationships among Late Pennsylvanian plant assemblages: palynological evidence from the markley formation. West Texas, U.S.A. rev. *Palaeobot. Palynol.* 211 10–27. 10.1016/j.revpalbo.2014.09.007 26028779 PMC4443493

[B41] Luginbuehl L. H. Menard G. N. Kurup S. Van Erp H. Radhakrishnan G. V. Breakspear A. (2017). Fatty acids in arbuscular mycorrhizal fungi are synthesized by the host plant. *Science* 356:1175. 10.1126/science.aan0081 28596311

[B42] Mander L. Kurschner W. M. Mcelwain J. C. (2010). An explanation for conflicting records of Triassic–Jurassic plant diversity. *Proc. Natl. Acad. Sci. U S A.* 107 15351–15356. 10.1073/pnas.1004207107 20713737 PMC2932585

[B43] Mandl K. Cantelmo C. Gruber E. Faber F. Friedrich B. Zaller J. G. (2018). Effects of glyphosate-, glufosinate- and flazasulfuron-based herbicides on soil microorganisms in a vineyard. *Bull. Environ. Contam. Toxicol.* 101 562–569. 10.1007/s00128-018-2438-x 30229276 PMC6223855

[B44] Martin F. Aerts A. Ahren D. Brun A. Danchin E. G. Duchaussoy F. (2008). The genome of *Laccaria* bicolor provides insights into mycorrhizal symbiosis. *Nature* 452 88–92. 10.1038/nature06556 18322534

[B45] Martin F. Uroz S. Barker D. G. (2017). Ancestral alliances: plant mutualistic symbioses with fungi and bacteria. *Science* 356:eaad4501. 10.1126/science.aad4501 28546156

[B46] Nicholas K. B. Nicholas H. B. (1997). GeneDoc: a Tool for Editing and Annotating Multiple Sequence Alignments. Pittsburgh: Pittsburgh Supercomputing Center’s National Resource for Biomedical Supercomputing.

[B47] Pan K. Zhao N. Yin Q. Zhang T. Xu X. Fang W. (2014). Induction of a laccase Lcc9 from *Coprinopsis cinerea* by fungal coculture and its application on indigo dye decolorization. *Bioresour. Technol.* 162 45–52. 10.1016/j.biortech.2014.03.116 24736211

[B48] Parniske M. (2008). Arbuscular mycorrhiza: the mother of plant root endosymbioses. *Nat. Rev. Microbiol.* 6 763–775. 10.1038/nrmicro1987 18794914

[B49] Parrent J. L. James T. Y. Vasaitis R. Taylor A. F. (2009). Friend or foe? evolutionary history of glycoside hydrolase family 32 genes encoding for sucrolytic activity in fungi and its implications for plant-fungal symbioses. *BMC Evol. Biol.* 9:148–148. 10.1186/1471-2148-9-148 19566942 PMC2728104

[B50] Phillips J. M. Hayman D. S. (1970). Improved procedures for clearing roots and staining parasitic and vesicular-arbuscular mycorrhizal fungi for rapid assessment of infection. *Trans. Brit. Mycol. Soc.* 55 158–161. 10.1016/S0007-1536(70)80110-3

[B51] Plumb R. S. Granger J. H. Stumpf C. L. Johnson K. A. Smith B. W. Gaulitz S. (2005). A rapid screening approach to metabonomics using UPLC and oa-TOF mass spectrometry: application to age, gender and diurnal variation in normal/Zucker obese rats and black, white and nude mice. *Analyst* 130 844–849. 10.1039/b501767j 15912231

[B52] Reinders A. Ward J. M. (2001). Functional characterization of the alpha-glucoside transporter sut1p from *Schizosaccharomyces pombe*, the first fungal homologue of plant sucrose transporters. *Mol. Microbiol.* 39 445–454. 10.1046/j.1365-2958.2001.02237.x 11136464

[B53] Rimington W. R. Pressel S. Duckett J. G. Bidartondo M. I. (2015). Fungal associations of basal vascular plants: reopening a closed book? *New Phytol.* 205 1394–1398. 10.1111/nph.13221 25537078

[B54] Rimington W. R. Pressel S. Duckett J. G. Field K. J. Bidartondo M. I. (2019). Evolution and networks in ancient and widespread symbioses between *Mucoromycotina* and liverworts. *Mycorrhiza* 29 551–565. 10.1007/s00572-019-00918-x 31720838 PMC6890582

[B55] Rodrigues A. Passarini M. R. Ferro M. Nagamoto N. S. Forti L. C. Bacci M. (2014). Fungal communities in the garden chamber soils of leaf-cutting ants. *J. Basic Microbiol.* 54 1186–1196. 10.1002/jobm.201200458 23681670

[B56] Roth R. Paszkowski U. (2017). Plant carbon nourishment of *Arbuscular mycorrhizal* fungi. *Curr. Opin. Plant Biol.* 39 50–56. 10.1016/j.pbi.2017.05.008 28601651

[B57] Ruan Y. (2014). Sucrose metabolism: gateway to diverse carbon use and sugar signaling. *Annu. Rev. Plant Biol.* 65 33–67. 10.1146/annurev-arplant-050213-040251 24579990

[B58] Sanderson M. J. (2003). r8s: inferring absolute rates of molecular evolution and divergence times in the absence of a molecular clock. *Bioinformatics* 19 301–302. 10.1093/bioinformatics/19.2.301 12538260

[B59] Sievers F. Wilm A. Dineen D. Gibson T. J. Karplus K. Li W. (2011). Fast, scalable generation of high-quality protein multiple sequence alignments using Clustal Omega. *Mol. Syst. Biol.* 7 539–539. 10.1038/msb.2011.75 21988835 PMC3261699

[B60] Spatafora J. W. Chang Y. Benny G. L. Lazarus K. Smith M. E. Berbee M. L. (2016). A phylum-level phylogenetic classification of zygomycete fungi based on genome-scale data. *Mycologia* 108 1028–1046. 10.3852/16-042 27738200 PMC6078412

[B61] Stacklies W. Redestig H. Scholz M. Walther D. Selbig J. (2007). pcaMethods—a bioconductor package providing PCA methods for incomplete data. *Bioinformatics* 23 1164–1167. 10.1093/bioinformatics/btm069 17344241

[B62] Strullu-Derrien C. Kenrick P. Pressel S. Duckett J. G. Rioult J. Strullu D. G. (2014). Fungal associations in *Horneophyton ligneri* from the Rhynie Chert (c. 407 million year old) closely resemble those in extant lower land plants: novel insights into ancestral plant-fungus symbioses. *New Phytol.* 203 964–979. 10.1111/nph.12805 24750009

[B63] Sun Y. Reinders A. Lafleur K. R. Mori T. Ward J. M. (2010). Transport activity of rice sucrose transporters OsSUT1 and OsSUT5. *Plant Cell Physiol.* 51 114–122. 10.1093/pcp/pcp172 19965875 PMC2807175

[B64] Thimm O. Blasing O. Gibon Y. Nagel A. Meyer S. Kruger P. (2004). Mapman: a user-driven tool to display genomics data sets onto diagrams of metabolic pathways and other biological processes. *Plant J.* 37 914–939. 10.1111/j.1365-313X.2004.02016.x 14996223

[B65] van Wyk, N., Trollope K. M. Steenkamp E. T. Wingfield B. D. Volschenk H. (2013). Identification of the gene for [beta]-fructofuranosidase from ceratocystis moniliformis cmw 10134 and characterization of the enzyme expressed in saccharomyces cerevisiae. *BMC Biotechnol.* 13:1–14. 10.1186/1472-6750-13-100 24225070 PMC3880211

[B66] Vargas W. A. Crutcher F. K. Kenerley C. M. (2011). Functional characterization of a plant-like sucrose transporter from the beneficial fungus *Trichoderma virens*. regulation of the symbiotic association with plants by sucrose metabolism inside the fungal cells. *New Phytol.* 189 777–789. 10.1111/j.1469-8137.2010.03517.x 21070245

[B67] Vargas W. A. Mandawe J. C. Kenerley C. M. (2009). Plant-derived sucrose is a key element in the symbiotic association between *Trichoderma virens* and maize plants. *Plant Physiol.* 151 792–808. 10.1104/pp.109.141291 19675155 PMC2754623

[B68] Voets L. Goubau I. Olsson P. A. Merckx R. Declerck S. P. (2008). Absence of carbon transfer between *Medicago truncatula* plants linked by a mycorrhizal network, demonstrated in an experimental microcosm. *FEMS Microbiol. Ecol.* 65 350–360. 10.1111/j.1574-6941.2008.00503.x 18557940

[B69] Wahl R. Wippel K. Goos S. Kamper J. Sauer N. (2010). A novel high-affinity sucrose transporter is required for virulence of the plant pathogen *Ustilago maydis*. *PLoS Biol.* 8:e1000303. 10.1371/journal.pbio.1000303 20161717 PMC2817709

[B70] Waipara N. W. (1997). *A study of the Taxonomy and Pathogenicity of Mcrofungi in the Roots of Waikato Pasture Plants.* PhD thesis, Research Repository.

[B71] Wang H. Zhang Y. Zhou W. Noppol L. Liu T. (2018). Mechanism and enhancement of lipid accumulation in filamentous oleaginous microalgae *Tribonema minus* under heterotrophic condition. *Biotechnol. Biofuels* 11:328. 10.1186/s13068-018-1329-z 30559837 PMC6290495

[B72] Wang M. Zhang Q. (2009). Issues in using the WinRHIZO system to determine physical characteristics of plant fine roots. *Acta Ecologica Sinica.* 29 136–138. 10.1016/j.chnaes.2009.05.007

[B73] Wang S. Yokosho K. Guo R. Whelan J. Ruan Y. L. Ma M. F. (2019). The soybean sugar transporter GmSWEET15 mediates sucrose export from endosperm to early embryo. *Plant Physiol.* 188 2133–2141. 10.1104/pp.19.00641 31221732 PMC6670074

[B74] Wei F. Hong Y. Liu J. Yuan J. Fang W. Peng H. (2010). Gongronella sp. induces overproduction of laccase in Panus rudis. *J Basic Microbiol.* 50 98–103. 10.1002/jobm.200900155 20082372

[B75] Wieczorke R. Krampe S. Weierstall T. Freidel K. Hollenberg C. P. Boles E. (1999). Concurrent knock-out of at least 20 transporter genes is required to block uptake of hexoses in *Saccharomyces cerevisiae*. *FEBS Lett.* 464 123–128. 10.1016/S0014-5793(99)01698-1 10618490

[B76] Wipf D. Krajinski F. Van Tuinen D. Recorbet G. Courty P. (2019). Trading on the arbuscular mycorrhiza market: from arbuscules to common mycorrhizal networks. *New Phytol.* 223 1127–1142. 10.1111/nph.15775 30843207

[B77] You Y. Park J. M. Seo Y. G. Lee W. Kang M. Kim J. (2017). Distribution, characterization, and diversity of the endophytic fungal communities on korean seacoasts showing contrasting geographic conditions. *Mycobiology* 45 150–159. 10.5941/MYCO.2017.45.3.150 29138619 PMC5673510

[B78] Young N. D. Debelle F. Oldroyd G. E. Geurts R. Cannon S. B. Udvardi M. K. (2011). The medicago genome provides insight into the evolution of rhizobial symbioses. *Nature* 480 520–524. 10.1038/nature10625 22089132 PMC3272368

[B79] Zhou G. Peng C. Liu X. Chang F. Fang Z. (2020). Identification and immobilization of an invertase with high specific activity and sucrose tolerance ability of *Gongronella sp*. w5 for high fructose syrup preparation. *Front. Microbiol.* 11:633. 10.3389/fmicb.2020.00633 32328053 PMC7160231

[B80] Zhou Y. Qu H. Dibley K. E. Offler C. E. Patrick J. W. (2007). A suite of sucrose transporters expressed in coats of developing legume seeds includes novel ph-independent facilitators. *Plant J.* 49 75–764. 10.1111/j.1365-313X.2006.03000.x 17253986

