# Supplementary material for: Mucoromycotina Fungi Possess the Ability to Utilize Plant Sucrose as a Carbon Source: Evidence From Gongronella sp. w5
Source: Front Microbiol. 2021 Jan 13;11:591697. doi: 10.3389/fmicb.2020.591697 (PMC7874188; doi:10.3389/fmicb.2020.591697)
Supplement: Supplementary file 5 [file Table_1.DOC]

**Supplemental materials**

**Figure S1** Multivariate analysis (PCA) of metabolite and gene expression data. a, metabolite data in positive ion mode. b, metabolite data in negative ion mode. c, gene expression data of transcriptome.


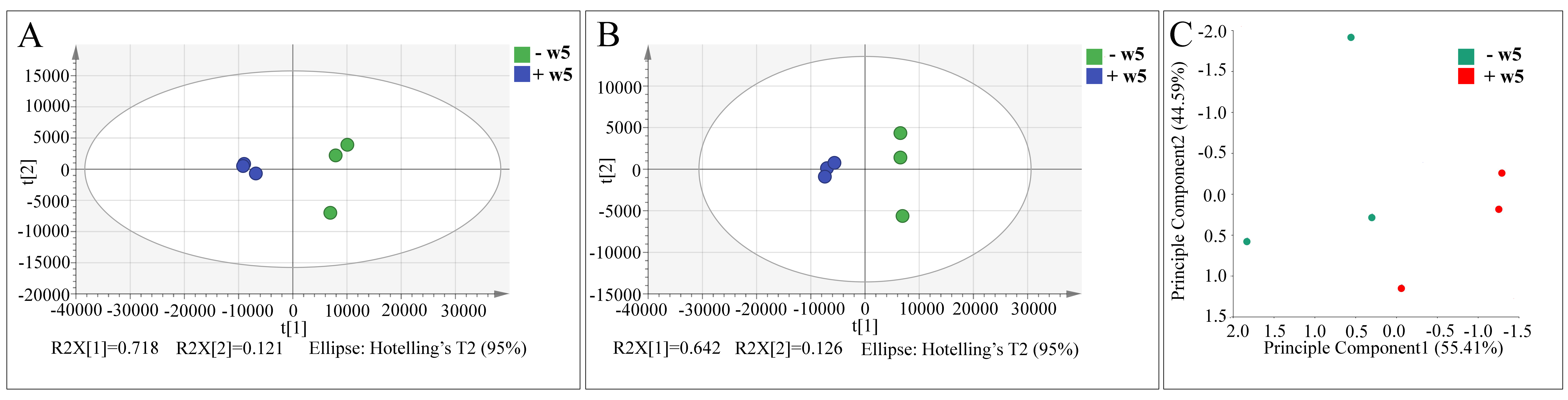


**Figure S2** Changes of metabolic pathways in root samples after inoculation with w5 at 16 dpi. (A) Top 10 enriched pathways from metabolomics data. (B) Top 10 enriched pathways from transcriptome data.


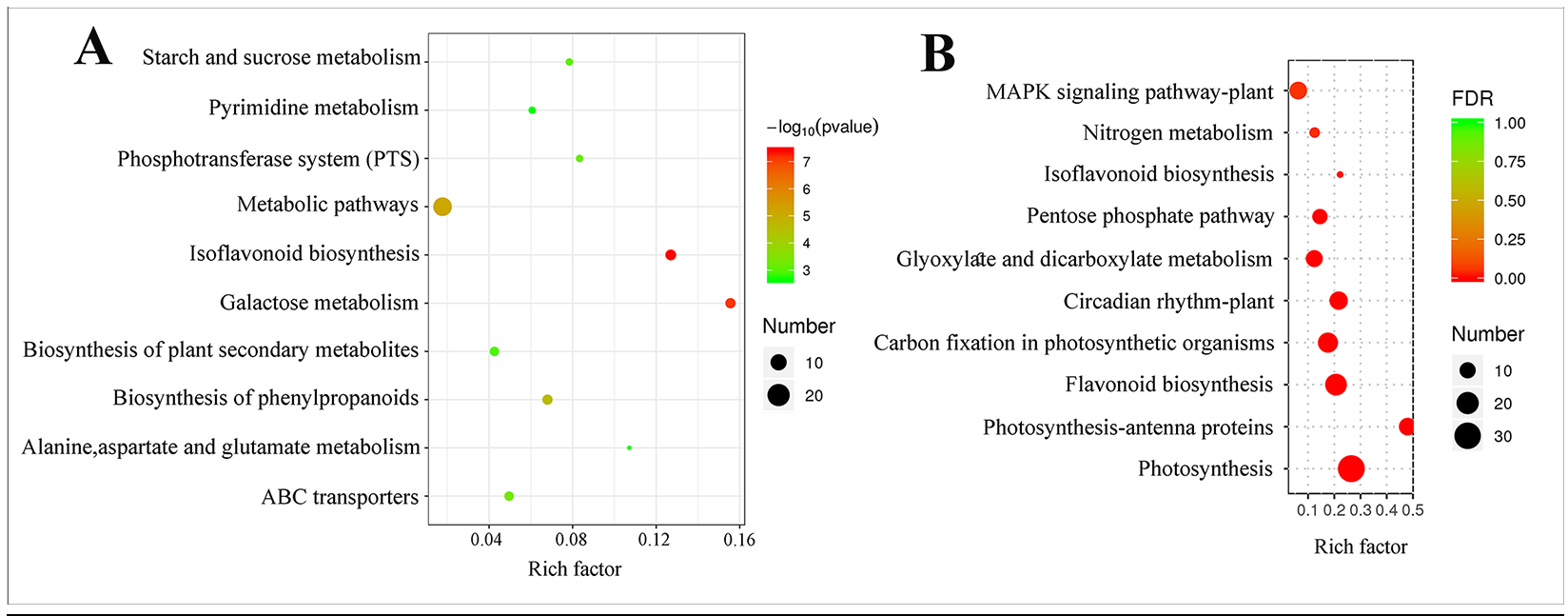


**Figure S3** Differential accumulation of metabolites and differential expression of related transcripts in *M. truncatula* roots after w5 inoculation.


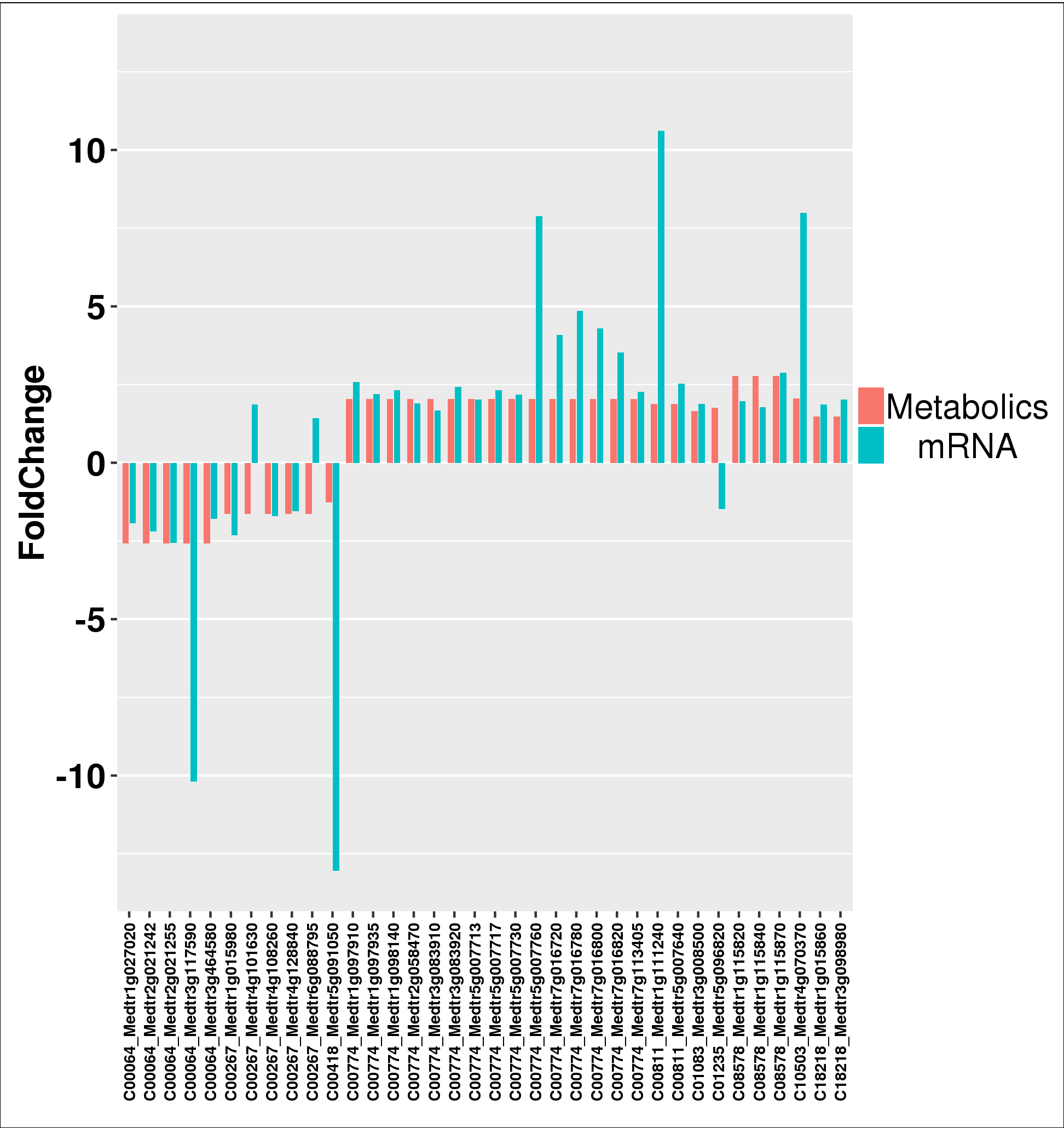


**Figure S4** Expression of secondary metabolic pathways-associated genes (a), cell wall (b), lipids (c), and hormone-associated genes (d) in *M. truncatula* roots of +w5 versus –w5.


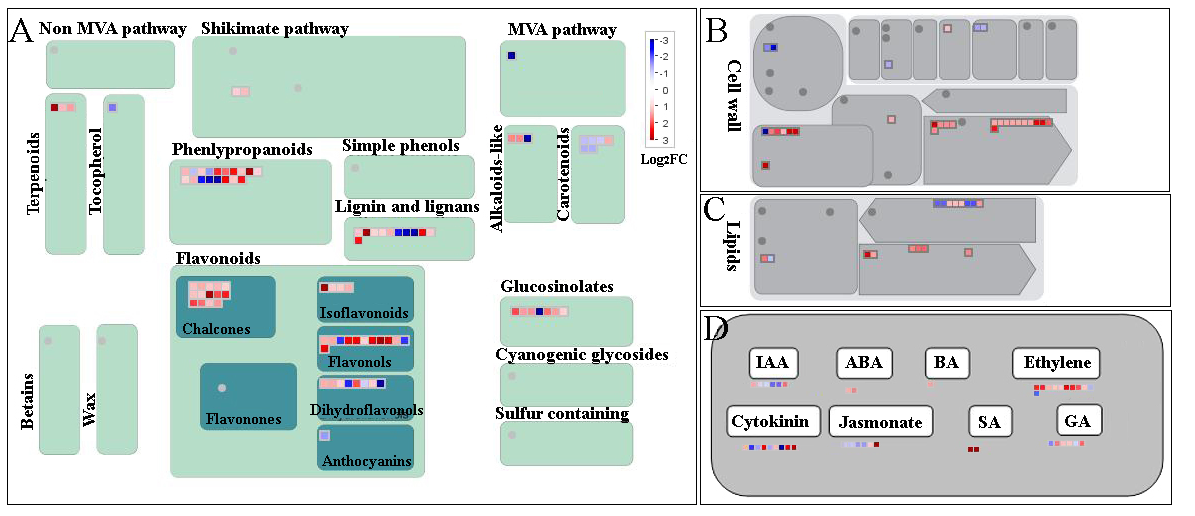


**Figure S5** Expression of sucrose and starch-associated genes in *M. truncatula roots* of +w5 versus –w5.


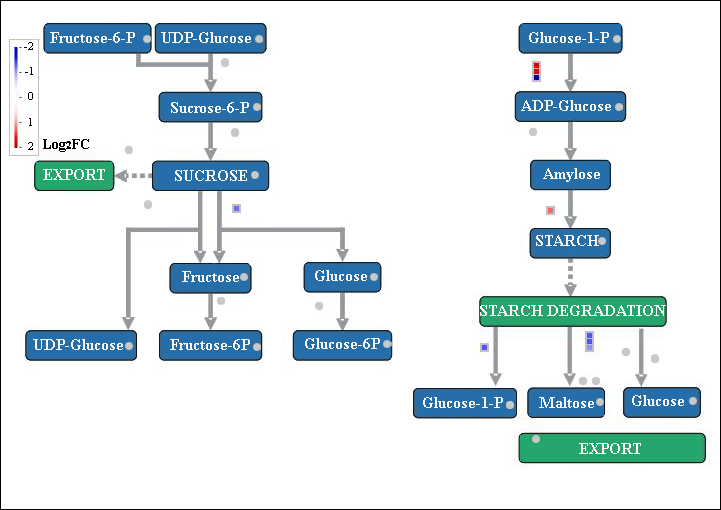


**Figure S6** Bioinformatic prediction of conserved domains A, D, and E of GH32 enzymes. Sequences from each fungus were aligned with Clustal Omega and logos were generated by using WebLogo. Amino acids of the catalytic site are denoted by asterisks.


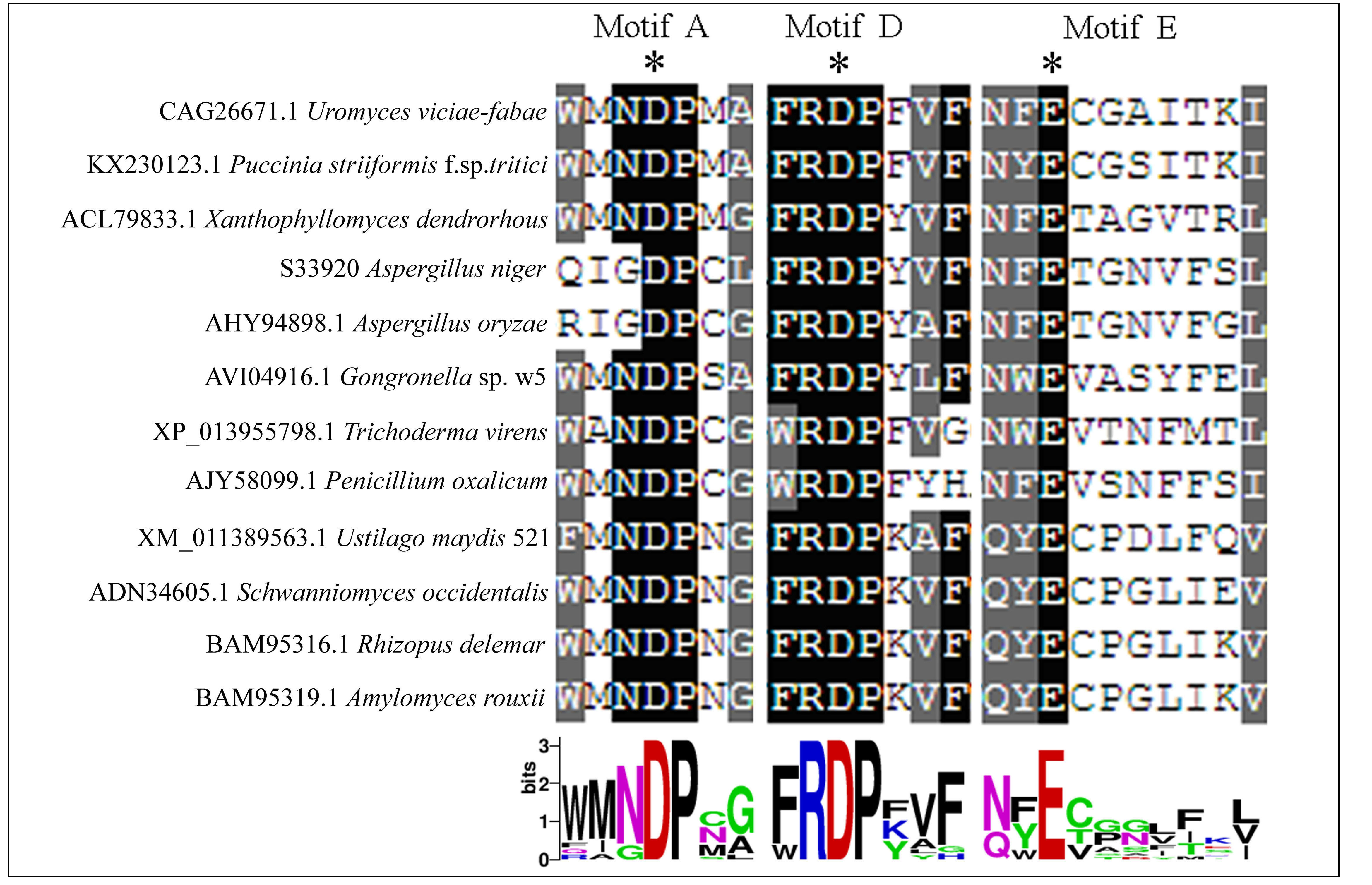


**Figure S7** Conserved domains of GspSUT1 and GspSUT2. Sequences from each fungus were aligned with Clustal Omega.


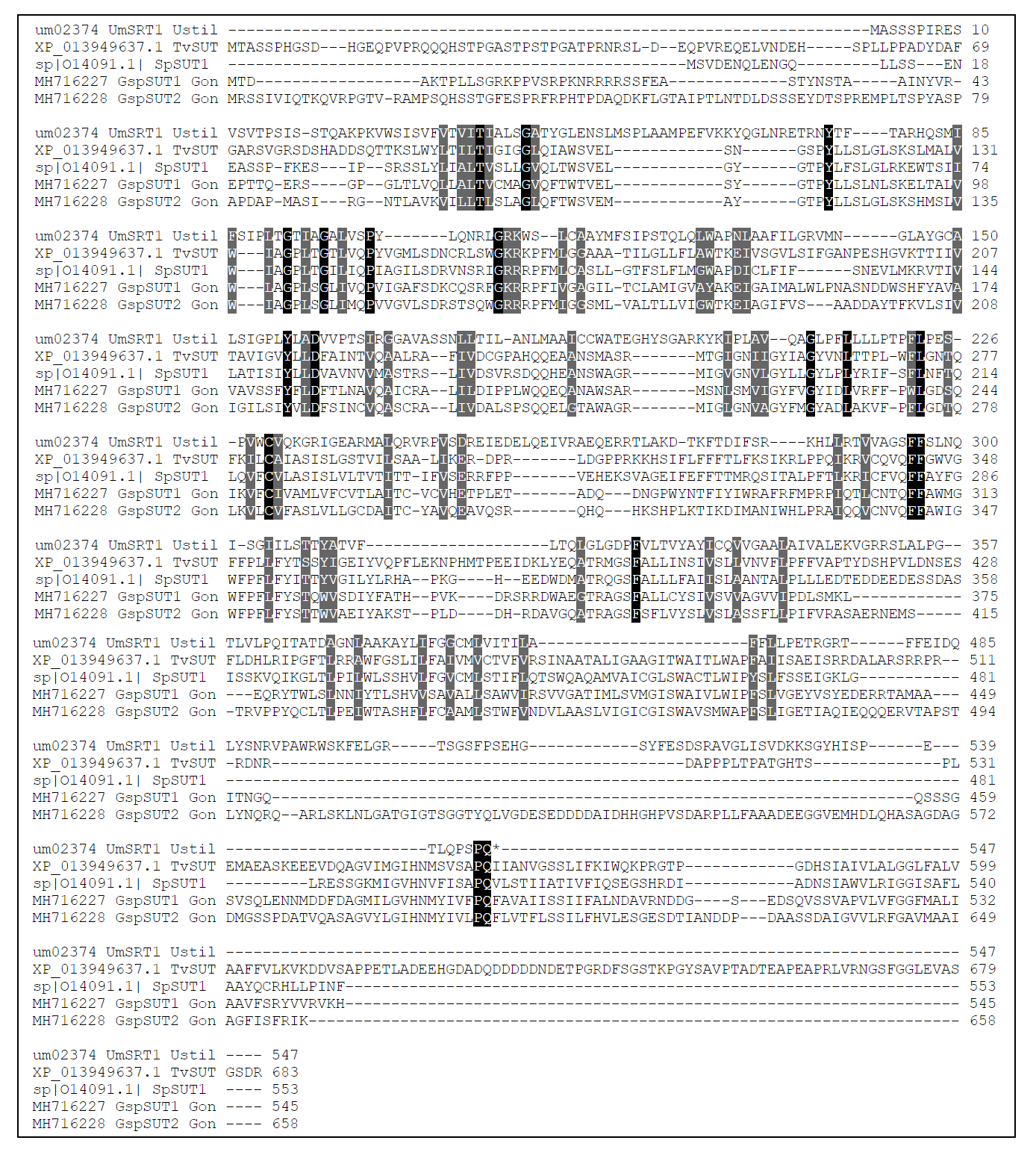


**Figure S8** A putative schematic overview of mechanism that *Gongronella* sp. w5 promotes *M. truncatula* growth by utilizing sucrose as the carbon source. Dashed lines with arrows indicate speculation.


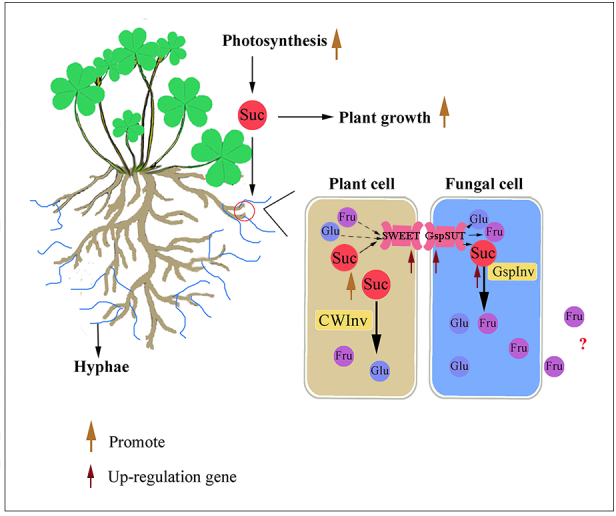


**Table S1** Sequences of primers used in qRT-PCR analysis.

| Primers | Nucleotide sequence (5’–3’) | Note |
| --- | --- | --- |
| GspINV-f1 | CCCTCGAGATGGTGCTTGCTGATCC | [KY817117](https://www.ncbi.nlm.nih.gov/nucleotide/KY817117.1?report=genbank&log$=nucltop&blast_rank=1&RID=VHZ4TCWK014); invertase gene of w5 for complementation of the yeast invertase defective strain SEY2102 |
| GspINV-r1 | TCCCCGCGGTCAAGGGCGATTGAACG |
| GAPDH1-f | CTTCCGTGTCCCTAAGCCTG | Reference gene of w5 for qRT-PCR |
| GAPDH1-f | CGACGTCATCCTCGGTGTAG |
| GAPDH2-f | GTGTCTTCACCACCACGGAT |
| GAPDH2-r | TCGAGATTGACACCGCAGAC |
| GspINV-f3 | TGATCACAGGCTTGATGCGT | AVI04916.1; invertase gene of w5 for qRT-PCR |
| GspINV-r3 | TCAAGGCAAGACGACGTTGA |
| Pma-GspSUT1-f | TTGCGGCCGCATGACCGACGCAAAGACACCGTT | MH716227; GspSUT1 gene of w5 for complementation of the yeast hexose transporter defective strain EBY.VW4000 |
| Pma-GspSUT1-r | TTGCGGCCGCTTAATGCTTGACACGCACG |
| Pma-MtSWEET15.3-f | TTGCGGCCGCATGGCCATTAGTCACAACAC | MtSWEET15.3 gene of *M. truncatula* for complementation of the yeast hexose transporter defective strain EBY.VW4000 |
| Pma-MtSWEET15.3-r | TTGCGGCCGCTTAAACTCCACAACCGATAGG |
| pDR-GspSUT1-f | CCCTCGAGATGACCGACGCAAAG | GspSUT1 gene of w5 for complementation of the yeast invertase defective strain SEY2102 |
| pDR-GspSUT1-r | TCCCCGCGGTTAATGCTTGACACGCACG |
| GspSUT1-f | GCGTTCAGCGTAAAGTCCA | Sucrose transporter genes of w5 for qRT-PCR |
| GspSUT1 -r | GGTTGCCCAATGCCTCC |
| GspSUT2-f | GCTGGGCGGATGATTG |
| GspSUT2-r | ACGCAGAGCACCTTGAGTT |
| MtEF-1α-F | GAGACCCACAGACAAGCC | Reference gene of *M. truncatula* for qRT-PCR |
| MtEF-1α-R | ACTGGCACAGTTCCAATACC |
| Medtr3g089125-f | CGGTCTGCTAGGCCGTGACC | SWEET transporter genes of *M. Truncatula* for qRT-PCR.  *MtSWEET15.3* (Medtr7g405730) and *MtSWEET1.2* (Medtr3g089125). |
| Medtr3g089125-r | CCCCTTCTGTTGGTTCCTGCTGT |
| Medtr7g405730-f | GCCGTGCATGGTCCACTTCG |
| Medtr7g405730-r | AGCCACAATGCTTAGTGGTGCTG |
| Medtr4g131920-f | CGCGGGAGCGTTGGGACTAA | Sucrose transporter geness of *M. Truncatula* for qRT-PCR.  *MtSUT1.2* (Medtr6g043880), *MtSUT1.3* (Medtr4g131920), *MtSUT1.4* (Medtr6g033580), *MtSUT2* (Medtr8g468330), *MtSUT4.1* (Medtr5g067470), and *MtSUT4.2* (Medtr3g110880). |
| Medtr4g131920-r | GCTCCGCCAATGAAACGCCC |
| Medtr8g468330-f | AAGGGACCCGAACAAGGGCTG |
| Medtr8g468330-r | TGGCCCCTGCACTGTGTTGTT |
| Medtr5g067470-f | TCGCATCCGTAGCAAGCGGT |
| Medtr5g067470-r | CGGAGACCGGTCCACAGAGC |
| Medtr3g110880-f | ACCGTCAACACCACCGGTCT |
| Medtr3g110880-r | GGCGACGGATGCTACTCGGA |
| Medtr6g033580-f | ACCGTGCCGTGCTTTCCTTG |
| Medtr6g033580-r | ATCCGGCAGCATACCCGAGG |
| Medtr6g043880-f | CCCACATCAGTGGGCTGCCA |
| Medtr6g043880-r | AGGGCGACGACGTCCGAAAC |
| Medtr2g099950-f | TGTGTGGGCCCATTCAGCATCA | Invertase genes of *M. truncatula* for qRT-PCR.  Medtr2g099950 (MtCWInv1), Medtr1g015980 (MtCWInv2) and Medtr8g017410 (MtCWInv3). |
| Medtr2g099950-r | TGCTGGTTTGCCACCTGGAAGT |
| Medtr1g015980-f | CCCTGATGGAGCAAGCTTTGGTG |
| Medtr1g015980-r | TGTGGCTGAGCCTGAGAAGCA |
| Medtr8g017410-f | ACAGTGGCCAGTTGTGGAAGTTGA |
| Medtr8g017410-r | ACTTGTGCTGCTGTGACACCATT |

**Table S2** Differentially expression of six randomly selected genes in *M. truncatula* roots +w5 versus –w5 validated by qRT-PCR

| Primers | Nucleotide sequence (5’–3’) | Putative annotation | RNA-Seq  (Log_2_Fold Change) | **p (corr)** | qRT-PCR  (Log_2_Fold Change) |
| --- | --- | --- | --- | --- | --- |
| Medtr1g111510-f | CGTCCAATTCATCACCGGGCG | MFS transporter | 1.39 | 1.2640E-4 | 1.06 |
| Medtr1g111510-r | ACATGTAGGTGGCTCCGGCTG |
| Medtr2g020710-f | GCACCCACTCAAGCGGCTATC | sugar porter (SP) family MFS transporter | 1.26 | 0.0253 | 1.10 |
| Medtr2g020710-r | TCTCATAGCCCCTTTCCTGCCA |
| Medtr1g111240-f | AGAAGCCGTTTGGCACAGAGT | cytochrome P450 family cinnamate 4-hydroxylase | 3.41 | 1.8658E-19 | 2.86 |
| Medtr1g111240-r | TGCCAGCCTCCTAGTTTGCAG |
| Medtr6g092620-f | AGCTATGGGCACAAAAGTGGCA | 1-aminocyclopropane-1-carboxylate oxidase | 2.51 | 9.5790E-14 | 2.62 |
| Medtr6g092620-r | CCACCAGCATCTGTATGCTCTCT |
| Medtr2g437380-f | ACTGCTTGTGAGGAGTGGGGA | 2OG-Fe(II) oxygenase family oxidoreductase | 2.47 | 1.6238E-12 | 2.64 |
| Medtr2g437380-r | GACTGTTCCTGGAGCCATAGGGT |
